# Upregulated Lipid Biosynthesis at the Expense of Starch Production in Potato (*Solanum tuberosum*) Vegetative Tissues *via* Simultaneous Downregulation of *ADP-Glucose Pyrophosphorylase* and *Sugar Dependent1* Expressions

**DOI:** 10.3389/fpls.2019.01444

**Published:** 2019-11-12

**Authors:** Xiaoyu Xu, Thomas Vanhercke, Pushkar Shrestha, Jixun Luo, Sehrish Akbar, Christine Konik-Rose, Lauren Venugoban, Dawar Hussain, Lijun Tian, Surinder Singh, Zhongyi Li, Peter J. Sharp, Qing Liu

**Affiliations:** ^1^Research Program of Traits, CSIRO Agriculture and Food, Canberra, ACT, Australia; ^2^Plant Breeding Institute and Sydney Institute of Agriculture, School of Life and Environmental Sciences, The University of Sydney, Sydney, NSW, Australia

**Keywords:** *Solanum tuberosum*, potato, RNA interference, *ADP-glucose pyrophosphorylase*, sugar dependent1, triacylglycerol

## Abstract

Triacylglycerol is a major component of vegetable oil in seeds and fruits of many plants, but its production in vegetative tissues is rather limited. It would be intriguing and important to explore any possibility to expand current oil production platforms, for example from the plant vegetative tissues. By expressing a suite of transgenes involved in the triacylglycerol biosynthesis, we have previously observed substantial accumulation of triacylglycerol in tobacco (*Nicotiana tabacum*) leaf and potato (*Solanum tuberosum*) tuber. In this study, simultaneous RNA interference (RNAi) downregulation of *ADP-glucose pyrophosphorylase* (AGPase) and *Sugar-dependent1* (SDP1), was able to increase the accumulation of triacylglycerol and other lipids in both wild type potato and the previously generated high oil potato line 69. Particularly, a 16-fold enhancement of triacylglycerol production was observed in the mature transgenic tubers derived from the wild type potato, and a two-fold increase in triacylglycerol was observed in the high oil potato line 69, accounting for about 7% of tuber dry weight, which is the highest triacylglycerol accumulation ever reported in potato. In addition to the alterations of lipid content and fatty acid composition, sugar accumulation, starch content of the RNAi potato lines in both tuber and leaf tissues were also substantially changed, as well as the tuber starch properties. Microscopic analysis further revealed variation of lipid droplet distribution and starch granule morphology in the mature transgenic tubers compared to their parent lines. This study reflects that the carbon partitioning between lipid and starch in both leaves and non-photosynthetic tuber tissues, respectively, are highly orchestrated in potato, and it is promising to convert low-energy starch to storage lipids *via* genetic manipulation of the carbon metabolism pathways.

## Introduction

Oil and fats, in the major form of triacylglycerols (TAGs), are one of the most energy-dense compounds in nature (Sanjaya et al., 2011). However, vegetable oil is mostly produced in the seeds or fruits; only a few vegetative tissues (*e.g. Cyperus esculentus*) have significant levels (Rahman et al., 2016). This is because most plant species rely on photosynthesis for carbon assimilation (O’Leary, 1988), from which the carbohydrate remains the typical carbon reservoir in vegetative tissues (Stitt, 1995), whereas TAG synthesized concomitantly is usually regarded as a byproduct of starch production (Chapman et al., 2013). Recently, a series of genetic engineering approaches have explored the potential of TAG production in plant biomass tissues mostly focused on the leaf, in attempts to exploit more sustainable and reliable vegetable oil production platforms (Xu and Shanklin, 2016; Xu et al., 2018; Vanhercke et al., 2019). It was revealed that through manipulating critical metabolic nodes involved in TAG metabolism, this product naturally at low levels could be remarkably accumulated in plant vegetative tissues without severely compromising plant development (Vanhercke et al., 2014; Vanhercke et al., 2017).

As reported in most recent studies, enhancement of TAG biosynthesis in plant vegetative tissues is usually accompanied by a reduction in starch accumulation (Vanhercke et al., 2014; Zale et al., 2016; Liu et al., 2017; Vanhercke et al., 2018). It was hypothesized that the carbon competition from starch is the dominant factor contributing to the boost of TAG accumulation in transgenic plants (Sanjaya et al., 2011). It is well known that pyruvate, the direct carbon source for the plastid *de novo* fatty acids biosynthesis, is derived from cytosolic glycolysis, which also supports starch production in the plastid (Rawsthorne, 2002; Zeeman et al., 2010; Chapman and Ohlrogge, 2012), so it still remains to be further investigated how the oil increase is compensated by the starch biosynthesis even with the same carbon source. Potato (*Solanum tuberosum*), which is the current 4^th^ largest staple food in the world and produces considerable amount of starch in the stolon tubers for both energy deposition and propagation (Levy et al., 2013; Pinhero et al., 2016; Zaheer and Akhtar, 2016), may provide a good platform to study the interrelationship between lipids and starch biosynthesis.

Mitchell et al. (2017) first investigated the starch quality and multiple nutritional properties in the high oil transgenic potato tubers overexpressing *WRINKLED1* (*WRI1*) *- DIACYLGLYCEROL ACYLTRANSFERASE 1* (*DGAT1*)*— OLEOSIN* genes under a tuber-specific manner. It was found that the starch amylose content and peak viscosity were significantly reduced, while gelatinization temperature was increased in the starch isolated from the transgenic tubers rich in oil compared to the wild type (WT). However, the transgenic potato, which showed the highest TAG accumulation of 3.3% on a tuber dry weight (DW) basis (almost 100-fold increase relative to WT), was generated through the enhancement of the TAG biosynthetic pathway, while the starch metabolism was not manipulated (Liu et al., 2017). It would be therefore of importance to further explore the intrinsic connections between starch and lipid in potato tubers concerning such a correlation reflected in the high oil transgenic potato. For example, the oil enhancement may also be realized through engineering the diversion of carbon flux from the starch biosynthesis, or suppressing the lipase activity.

Presently, metabolic pathways regulating starch anabolism and TAG catabolism in plants are basically elucidated (Zeeman et al., 2010; Tjellström et al., 2015). In potato tuber, a series of enzymes are involved in starch biosynthesis, such as the *AGPase*, granule-bound starch synthase (*GBSS*), starch synthase II (*SSII*) and starch branching enzyme (*SBE*) (Schwall et al., 2000; Jobling et al., 2002; Grommers and van der Krogt, 2009). Among these, *AGPase* is the key rate-limiting enzyme initiating starch biosynthesis, and could therefore be manipulated to regulate the carbon flux (Geigenberger, 2003; Tetlow et al., 2004; Hwang et al., 2007; Jonik et al., 2012). The *SDP1* gene, which was reported to account for over 95% of TAG turnover by disintegrating the lipid droplets (LD) in plants (Kelly and Feussner, 2016), is thought to be the primary lipase for oil degradation (Scherer et al., 2010; Fan et al., 2014; Thazar-Poulot et al., 2015).

Studies on *AGPase* in terms of oil accumulation were mainly reported in algae, from which the downregulation of *AGPase* was able to boost TAG production (Radakovits et al., 2010; Li et al., 2010; Siaut et al., 2011), which was similar to an Arabidopsis (*Arabidopsis thaliana*) mutant deficient in starch (Yu et al., 2018). However, a similar result was not observed in potato tubers by repressing the same gene as the content of total fatty acids was barely changed despite considerable reduction in starch accumulation (Klaus et al., 2004). This suggests the possible necessity to co-regulate *AGPase* together with other genes involved in the lipid metabolism in order to enhance lipids accumulation in potato tubers. Indeed, it was later revealed that the inhibition of *AGPase* expression, together with the ectopic overexpression of *WRI1* had cooperatively boosted TAG accumulation in Arabidopsis leaves (Sanjaya et al., 2011). A similar result was also reported in transgenic sugarcane (*Saccharum officinarum*), where downregulation of *AGPase* and manipulation of several other genes involved in TAG biosynthesis and fatty acid β-oxidation dramatically increased TAG content (Zale et al., 2016). Likewise, in terms of the lipase *SDP1*, Kelly et al. (2013) reported that the suppression of *SDP1* had increased the TAG content of Arabidopsis leaves, and was further enhanced when co-expressed with *WRI1* and *DGAT1*. By disrupting the expression of *SDP1* in a trigalactosyldiacylglycerol1-1 (*tgd1-1*) mutant of Arabidopsis, 9% TAG was produced on a leaf DW basis (Fan et al., 2014). Further, through RNAi downregulation of *SDP1* expression in the high oil transgenic tobacco (*Nicotiana tabacum*) expressing *AtWRI1-AtDGAT1-* sesame (*Sesamum indicum*) *OLEOSIN1* (*SiOLEOSIN1*), TAG accumulation was doubled from 15% to 30% of leaf DW compared to the original transgenic line (Vanhercke et al., 2017). These studies demonstrate the possibility to further enhance oil accumulation in plant vegetative tissues by diverting more carbon away from starch and towards lipid biosynthesis, whilst preventing TAG turnover from lipase activity. In contrast, the singular manipulation of either *AGPase* or *SDP1* was proven to be less effective.

In this study, the endogenous *StAGPase* and *StSDP1* genes of potato were downregulated with a duplex RNAi approach in both WT and the high-oil transgenic potato line known as HO69 with more than 3% TAG in the tuber. Through simultaneously manipulating the carbon flux in potato tubers from the “starch source” (Jonik et al., 2012) and ‘TAG sink’ (Du and Benning, 2016; Bozhkov, 2018), it was anticipated that the carbon partitioning between carbohydrate and lipid would be reconstituted. A series of biochemical and microscopic analyses were thereafter carried out to characterize the biosynthesis of lipids and starch in the transgenic potatoes. With a particular focus on the carbohydrate and lipid metabolisms, the conversion of relatively low-energy starch to the energy-dense storage lipids was realized in potato tubers through these genetic engineering approaches, which may provide insight and further advance our understanding of the carbon reallocation and equilibration in plant biomass tissues.

## Results

### Regeneration and Selection of Transgenic Potato Plants Expressing the RNAi Cassette Targeting the *StaGPase* and *StSDP1* Genes Simultaneously

WT potato and the HO69 were simultaneously transformed with a construct harboring a RNAi cassette simultaneously targeting the downregulations of *StAGPase* and *StSDP1* expression driven by the *CaMV-35S* promoter. A total of eleven and three independent T_0_ plants were obtained from the WT- and HO69-derived transformations respectively, as verified by polymerase chain reaction (PCR) amplification of a DNA fragment of the hygromycin-B-phosphotransferase (*HPH*) gene, which confers hygromycin resistance as the selectable marker during gene transformation, from developing leaves of the regenerated potato plants (**data not shown**).

**Figure 1:**
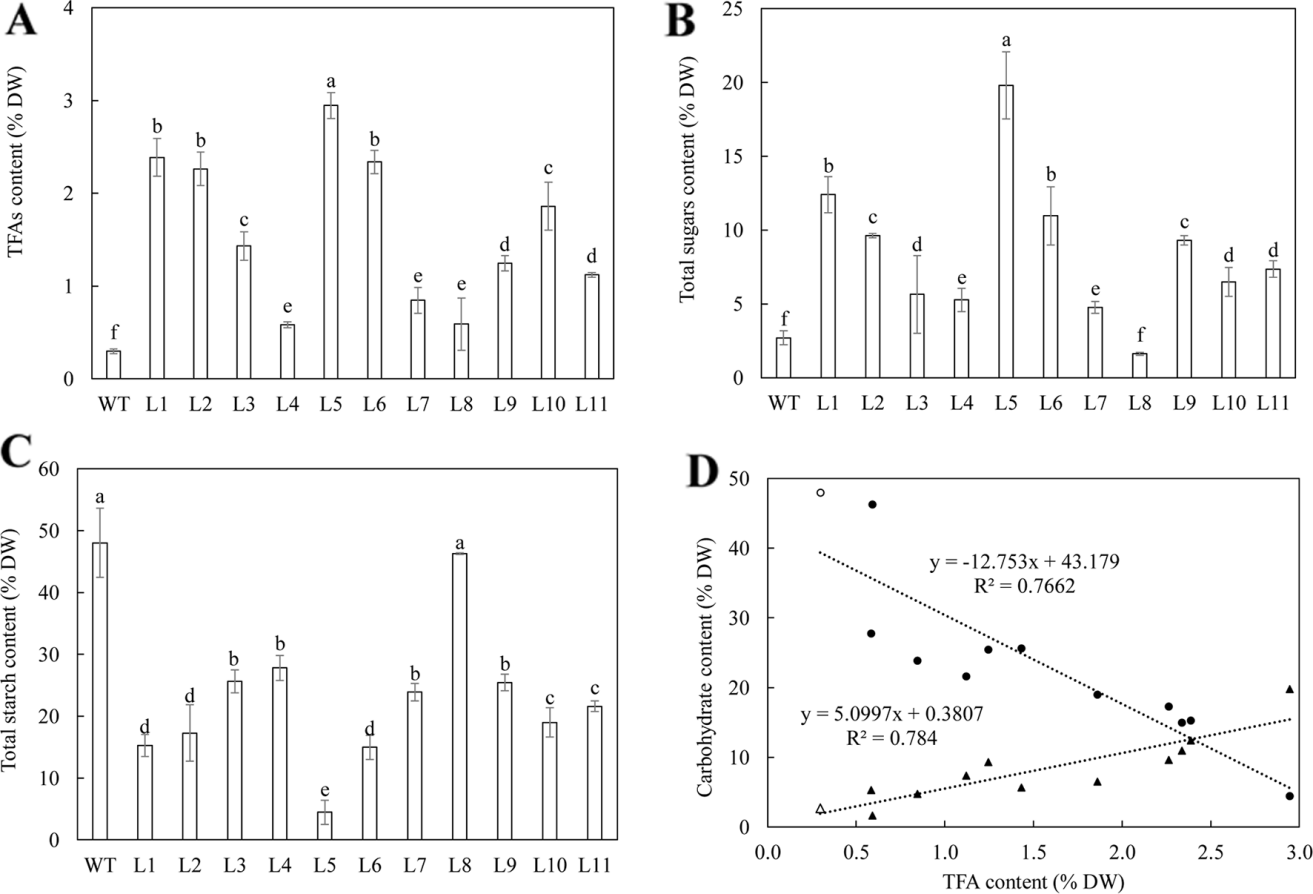
Contents of total fatty acids (TFA) and major carbohydrate in the mature potato tubers of WT and the eleven selected T_0_ generation plants. **(A)** TFA content; **(B)** Total soluble sugars content; **(C)** Total starch content; **(D)** Regression analysis among TFA, total soluble sugars (triangles) and starch (circles) in WT (open symbols) and transgenic lines (black symbols). The relationship between TFA and starch is negative (y = -12.753x + 43.179, R² = 0.7662), while the relationship between TFA and the total soluble sugars is positive (y = 5.0997x + 0.3807, R² = 0.784). The data represent the mean values ± standard deviation (SD) of three biological replicates. Letters (a, b, c, etc.) above the bars are all based on the least significant difference (LSD). Different letters between lines are statistically significantly different at P < 0.05.

Analysis of the contents of total fatty acids (TFA), starch and total soluble sugars in transgenic tubers were carried out for an overview of the RNAi effects. The eleven WT-derived lines showed 1.9 to 9.8-fold increase in the content of TFA (**Figure 1**), 1.7 to 7.3-fold increase in total soluble sugars content (**Figure 1**), and 1.7 to 10.8-fold reductions in starch content compared to WT (**Figure 1**). Regression analysis showed that the accumulation of TFA was negatively correlated with starch (a coefficient of -0.88), but positively correlated to total soluble sugars (a coefficient of 0.89); the contents of total soluble sugars and starch were negatively correlated (a coefficient of -0.86) (**Figure 1**).

Two transgenic lines, WT-L5 and WT-L10, with enhanced lipid accumulation relative to WT in mature tubers (0.3% of tuber DW), representing the highest level of TFA at 2.95% and a moderate level at 1.86% respectively, were selected for further analysis. In the HO69-derived super-transformation, due to a limited transgenic population, all the 3 super-transformed lines, named 69-L1, 69-L2, and 69-L3, were proceeded to synchronically propagate with HO69, WT, WT-L5, and WT-L10 for characterization at two developmental stages, the flowering stage and mature stage in a new generation.

### Downregulation of Target Genes and Alteration in the Accumulation of Lipids and Carbohydrate in the Leaves of WT-Derived Transgenic Lines

Assessments of *StAGPase* and *StSDP1* gene expression in the fully expanded leaves of WT and the two selected transgenic lines, WT-L5 and WT-L10, were carried out in both the flowering and mature stages (**Supplementary Figures 1A, B**). Significant downregulation of both these two genes were detected, with WT-L5 displaying the lowest expression levels at the mature stage (**Supplementary Figure 1B**). Alterations in the accumulation of carbohydrate and TFA were also observed. WT-L10 displayed significantly enhanced TFA production (6.61% of leaf DW) relative to WT at the mature stage, representing a 1.3-fold increase (**Supplementary Figure 1D**), while WT-L5 did not show substantial variation in both stages. The starch content of WT-L5 were lower at 1.99% and 0.8% of leaf DW at both the flowering stage and mature stage respectively, compared to 7.23% and 17.41% in WT at the respective stages. The content of total soluble sugars was increased to 11.49% at the flowering stage and 7.81% at the mature stage, representing 4.4-fold and 1.7-fold increase relative to WT, respectively (**Supplementary Figures 1C, D**). WT-L10 also exhibited a significant decrease in starch contents at the two developmental stages relative to WT, displaying a 1.3 and 1.1-fold reduction respectively, but not in total soluble sugars content, which was significantly increased in the flowering stage (**Supplementary Figure 1D**).

The TAG content in WT-L5 was not increased relative to WT over the two developmental stages, instead, a declining trend in TAG accumulation was observed (**Supplementary Figure 2A**), while in WT-L10, the accumulation of TAG peaked at 0.07% (almost a 2-fold increase) in the flowering stage, but dropped thereafter. Furthermore, WT-L5 did not show significant alteration in the contents of polar lipids relative to WT, whereas WT-L10 exhibited significantly decreased monogalactosyldiacylglycerol (MGDG) and digalactosyldiacylglycerol (DGDG) at the flowering stage (0.7% and 0.31% of leaf DW, respectively) (**Supplementary Figure 2B**), but subsequently increased to the maximum level at the mature stage (**Supplementary Figure 2C**). In particular, the galactolipids demonstrated an almost 2-fold increase in WT-L10 compared to WT, and the contents of the phospholipids including phosphotidylcholine (PC) and phosphatidylethanolamine (PE) were also significantly increased, but to a lesser extent. In contrast, the content of phosphatidylglycerol (PG) did not show significant variation among WT and the two transgenic lines. Significantly enhanced free fatty acids (FFA) accumulation was observed in WT-L10, but was reduced in WT-L5 compared to the WT at the mature stage, but not at the flowering stage (**Supplementary Figure 2D**).

The significant change in the proportion of C18 polyunsaturated fatty acids (PUFA) represented the major fatty acid variation in leaf between transgenic and WT potatoes (**Supplementary Figure 3**). At the flowering stage, TAG in the two selected lines showed significantly increased linoleic acid (LA, C18:2^Δ9,12^) and reduced α-linolenic acid (ALA, C18:3^Δ9,12,15^) levels relative to WT (**Supplementary Figure 3A**). Oleic acid (C18:1^Δ9^) and long chain fatty acids (LCFA, ≥ C20) were also increased in the two transgenic lines. Such a trend was persistent in WT-L5 in the mature stage, however WT-L10 showed significantly reduced LA but increased ALA levels relative to WT (**Supplementary Figure 3B**). Similarly, LA and ALA ratios of PC were altered in the two transgenic lines relative to WT (**Supplementary Figures 3C, D**). However, MGDG and DGDG did not show consistent variations in the fatty acid compositions in the two selected transgenic lines compared to WT, except for that WT-L5 showed significantly increased LA and decreased ALA at the mature stage (**Supplementary Figures 3F, H**).

### Enhancement in Lipid Accumulation at the Expense of Starch in the Tubers of WT-Derived Transgenic Lines

**Figure 2:**
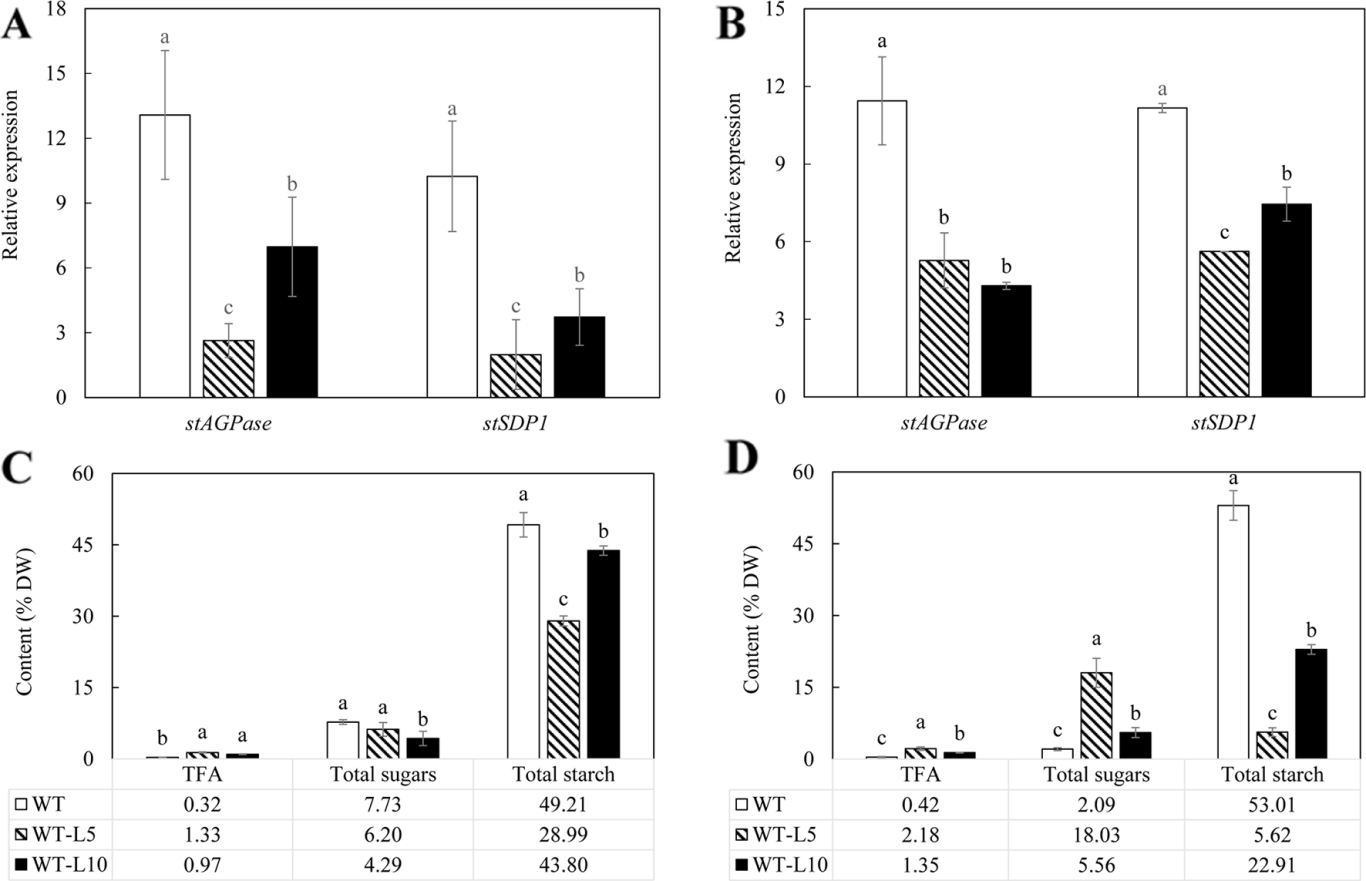
Gene expression analysis and total carbon allocation in the potato tubers of WT (open bars) and the two selected WT-derived lines, WT-L5 (hatched bars) and WT-L10 (black bars) at two developmental stages. **(A)** Real-time quantitative reverse transcription polymerase chain reaction (qRT-PCR) result at the flowering stage; **(B)** Real-time qRT-PCR result at the mature stage; **(C)** Total carbon allocation at the flowering stage; **(D)** Total carbon allocation at the mature stage. The data represent the mean values ± SD of three biological replicates. Letters (a, b, c) above the bars are based on LSD, bars marked with different letters are statistically significantly different at P < 0.05.

As shown in **Figure 2A**, expression of both *StAGPase* and *StSDP1* was significantly suppressed in the tubers of the two transgenic lines compared to WT, with WT-L5 displaying more severe downregulations than WT-L10 at the flowering stage. Both WT-L5 and WT-L10 had significantly enhanced accumulation of TFA at the flowering stage relative to WT (**Figure 2C**), which were further elevated to 2.18% and 1.35% of tuber DW, respectively, at the mature stage. Relative to WT, the TFA content was an approximately 8-fold higher in WT-L5, and 3.2-fold higher in WT-L10 (**Figure 2D**). A significant reduction in starch content was observed in WT-L5, which was almost 10-fold lower relative to WT at the mature stage (5.62%), accompanied by significantly increased accumulation of total soluble sugars (18.03%), which was 9-fold higher than that in WT (**Figure 2D**). Likewise, significant reduction in starch content and increase in total soluble sugars relative to WT were observed in WT-L10 over the two developmental stages, but to a lesser extent relative to WT-L5.

**Figure 3:**
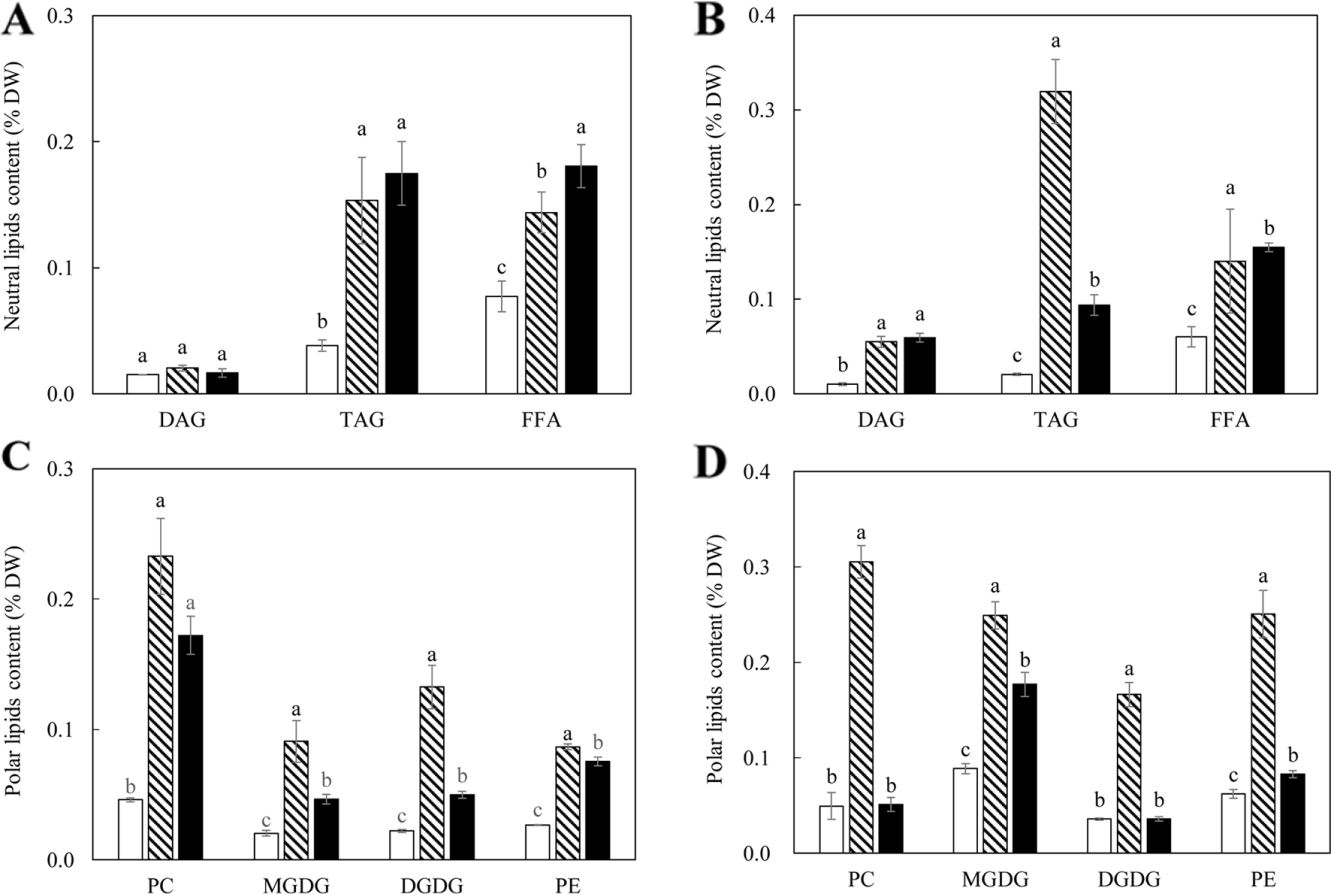
Contents of neutral and polar lipids in the potato tubers of WT (open bars) and the two selected WT-derived lines, WT-L5 (hatched bars) and WT-L10 (black bars) at two developmental stages. **(A)** Neutral lipids contents at the flowering stage; **(B)** Neutral lipids contents at the mature stage; **(C)** Polar lipids contents at the flowering stage; **(D)** Polar lipids contents at the mature stage. The data represent the mean values ± SD of three biological replicates. Letters (a, b, c) above the bars are based on LSD, bars marked with different letters are statistically significantly different at P < 0.05.

The contents of TAG, diacylglycerol (DAG), and FFA were further analyzed following fractionation through thin layer chromatography (TLC) (**Figures 3A, B**). TAG accumulation in the tubers of WT-L5 had significantly increased throughout the tuber development, which was 0.15% of tuber DW at the flowering stage, further increased to 0.32% at the mature stage, representing a 16-fold enhancement relative to WT in the mature stage (**Figure 3B**). In WT-L10, the TAG content in tubers peaked at the flowering stage as 0.17%, but reduced thereafter. The accumulations of DAG and FFA in transgenic tubers were also significantly increased compared to WT. For example, WT-L10 displayed 6-fold and almost 3-fold increase in DAG and FFA, respectively, at the mature stage (**Figure 3B**). Likewise, the significant increase in phospholipids was detected in the two transgenic lines. At the flowering stage, the contents of PC were increased 5 and 3-fold, respectively in WT-L5 and WT-L10 compared to WT, as well as in PE, where WT-L5 and WT-L10 showed 3- and 2.6-fold increases, respectively, compared to WT (**Figure 3C**). However, at the mature stage, significant increase in the accumulation of phospholipids was only observed in WT-L5, while it was dropped to the WT level in WT-L10 (**Figure 3D**). Significant increases in the contents of galactolipids were also observed in the two transgenic lines, particularly prominent in WT-L5, which showed nearly 5-fold increase in MGDG and 6- fold increase in DGDG relative to WT at the flowering stage (**Figure 3C**). At the mature stage, further increase of MGDG in WT-L10 have also been observed (**Figure 3D**).

The fatty acid composition of TAG in the tubers of WT-L5 and WT-L10 mainly showed perturbations in the saturated and monounsaturated fatty acids relative to WT at the flowering stage, while WT-L5 also displayed significantly reduced ALA level (**Supplementary Figure 4A**). In the mature stage, the two transgenic lines both showed similar fatty acid profiles, featured by significantly reduced LA and saturated fatty acids but increased ALA compared to WT (**Supplementary Figure 4B**). In contrast, PC exhibited a relatively constant fatty acid composition in the two transgenic lines over the two stages, demonstrating significantly increased LA but reduced ALA levels relative to WT (**Supplementary Figures 4C, D**). In MGDG and DGDG, the variation of fatty acid compositions was reflected by the significantly decreased saturated fatty acids and alteration in the ratio of C18 PUFAs in transgenic tubers, as exemplified by the significant increase in ALA at the expense of LA in MGDG in WT-L10 relative to WT at the flowering stage (**Supplementary Figure 4E**). However, the ratio of LA/ALA dropped significantly at the mature stage in both WT-L5 and WT-L10 (**Supplementary Figure 4F**).

### Starch Property Analysis of the Mature Potato Tubers in the WT-Derived Transgenic Lines

**Figure 4:**
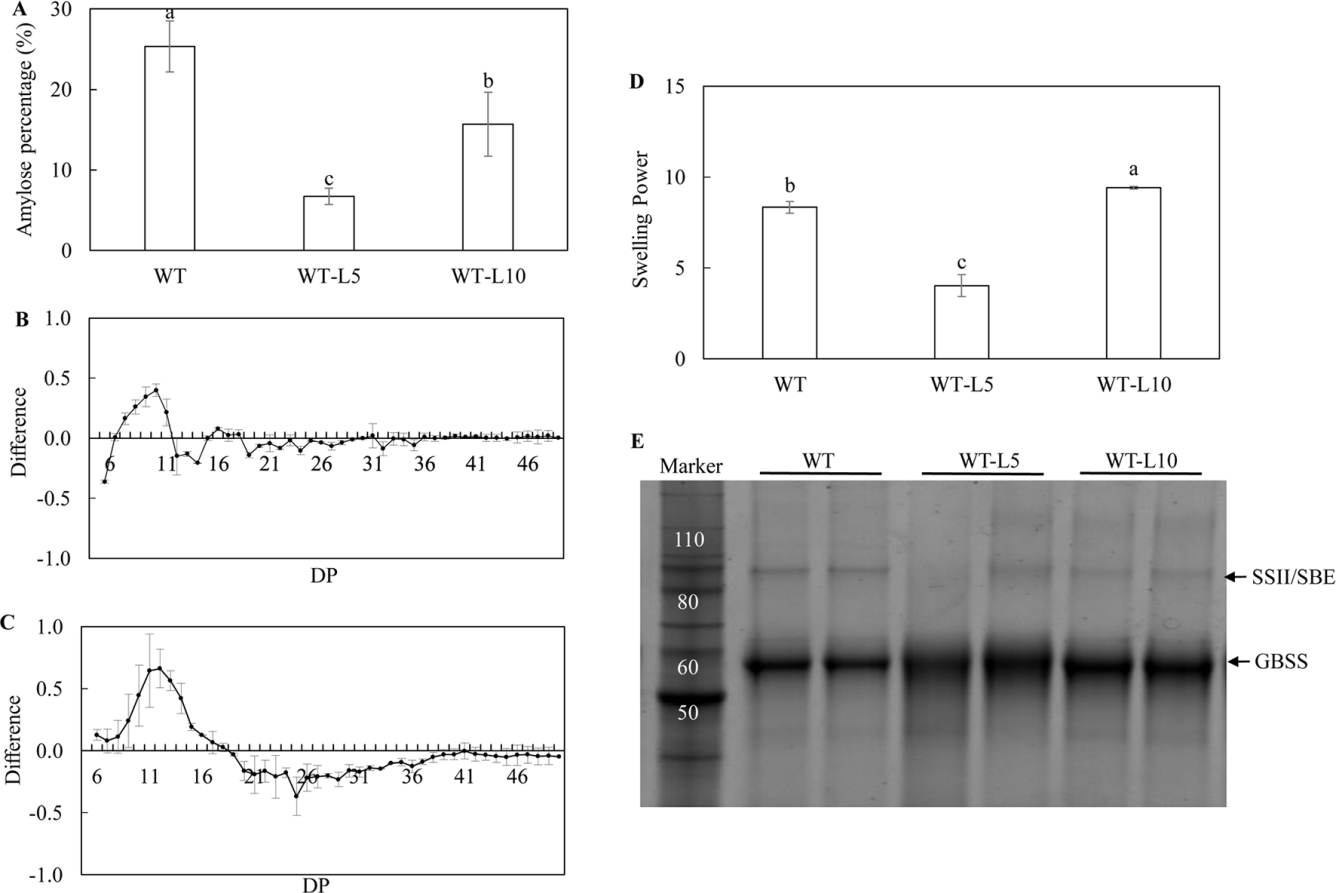
Analysis of the starch properties in the mature potato tubers. **(A)** Amylose content; **(B)** Chain length distribution (CLD) variation in debranched starch samples of WT-L5 relative to WT; **(C)** CLD variation in debranched starch samples of WT-L10 relative to WT; The CLD was reflected as the degree of polymerization (DP), each mark above the horizontal axis corresponds to the difference of a chain length in mole percentage. The error bars represent the standard errors, and the difference value was obtained by subtracting the CLD of WT from the two selected RNAi lines, respectively. **(D)** Swelling power of potato flour; **(E)** SDS-PAGE of potato starch granule bound proteins (GBPs). The data represent the mean values ± SD of three biological replicates. Letters (a, b, c) above the bars are based on least significant difference (LSD), bars marked with different letters are statistically significantly different at P < 0.05.

The biochemical analysis of starch properties, including the structure and functionality, was carried out using the starch isolated from mature potato tubers. Significant reduction in amylose content in the tubers of WT-L5 and WT-L10, relative to WT, was observed. In particular, the amylose contents in WT-L5 and WT-L10 tuber were approximately 4- and 2-fold lesser, respectively, than that in WT (**Figure 4A**). An altered chain length distribution (CLD), which was reflected as the degree of polymerization (DP) of the debranched starch was demonstrated. Compared to WT, both WT-L5 and WT-L10 showed a preference for the accumulation of short chains (DP < 11). In WT-L5, a decrease in the accumulation of intermediate chains between DP12 and DP16, although not statistically significant relative to WT, was observed (**Figure 4B**). In WT-L10, a tendency of significant reduction in the intermediate chains between DP12 and DP19 relative to WT, was observed (**Figure 4C**). As a result, the swelling power of the tuber flours of the two transgenic lines were altered differently. The swelling power of WT-L5 showed over 2-fold reduction compared to WT, whereas that of WT-L10 increased significantly (**Figure 4D**). Two main protein bands were identified in the SDS-PAGE gel, which were recognized as the granule bound proteins (GBPs) of the potato starch. According to the protein ladder standard, the potato GBSS band was localized near the size of 60 kDa and the SSII/SBE band was at the size of 90 kDa. Visual increase in the GBSS abundance relative to WT was observed in the two transgenic lines, while SSII/SBE reflected a visually discernible decrease in both the two transgenic lines (**Figure 4E**). The digital analysis of the protein band intensity on SDS gel revealed a consistent trend (**Supplementary Table 1**), the GBSS protein bands of WT-L5 and WT-L10 both displayed an average 1.2-fold enhancement compared to WT, while the SSII/SBE proteins showed 0.65- and 0.57-folds decrease of the band intensity, respectively.

### Suppression of Target Genes Expression and Alteration in Lipids and Carbohydrate in the Leaves of the HO69-Derived Super-Transformed Potato Lines

Downregulation of the *StAGPase* and *StSDP1* expression in the leaves of the three super-transformed potato lines was reflected over the two developmental stages. Although the RNAi targeted DNA fragments derived from *StAGPase* and *StSDP1* gene sequences were physically linked in the RNAi cassette, these two genes were differentially downregulated. For example, at the flowering stage, the strongest *StAGPase* suppression was observed in 69-L1 while the strongest *StSDP1* suppression was observed in 69-L3 (**Supplementary Figure 5A**). However, the downregulation levels of the two target genes were basically consistent among the three super-transformed lines at the mature stage (**Supplementary Figure 5B**).

Significant fluctuations of the carbon allocation between lipids and carbohydrate were displayed corresponding to the alteration in the expression levels of *StAGPase* and *StSDP1*. Up to 5.31% TFA (DW) was observed in the leaves of 69-L3 at the flowering stage, which was accompanied by a 2.5-fold increase in the total soluble sugars and significantly reduced starch relative to HO69 (**Supplementary Figure 5C**). However in 69-L1 and 69-L2, the variations were relatively less significant. At the mature stage, enhanced accumulation in the total soluble sugars were observed in 69-L1 (11.04%) and 69-L3 (7.19%), followed by a nearly 2-fold reduction in the starch content relative to HO69, while the TFA levels were not significantly varied (**Supplementary Figure 5D**).

Among the three super-transformed lines, 69-L3 displayed the highest accumulation of TAG at the flowering stage (1.84%), which was nearly 5-fold higher than HO69, while both 69-L1 and 69-L2 showed moderate yet significant increases (**Supplementary Figure 6A**). At the mature stage, all the three super-transformed lines showed almost doubled TAG accumulations compared to HO69. By comparison, most polar lipids in the three super-transformed lines remained unchanged relative to HO69 at the flowering stage, except that 69-L2 and 69-L3 displayed significant reduction in PC content, and increase in the content of DGDG in 69-L2 (**Supplementary Figure 6B**). At the mature stage, 69-L3 exhibited significant increase in the contents of PC (0.6%), MGDG (0.86%) and DGDG (0.52%) (**Supplementary Figure 6C**). Similar increases in the phospholipids were also observed in 69-L1, but not in 69-L2. The accumulations of FFAs, on the contrary, were all significantly decreased in the three super-transformed lines compared to HO69 over the two developmental stages (**Supplementary Figure 6D**).

Compared to HO69, the variation in the fatty acid compositions of TAG in the three super-transformed lines was mainly reflected in the levels of palmitic (C16:0) and oleic acids (**Supplementary Figures 7A, B**). Specifically, 69-L2 and 69-L3 showed significant reduction in palmitic acid over the two developmental stages, while the relative content of oleic acid was almost doubled at flowering stage (**Supplementary Figure 7A**). The fluctuations in the relative contents of other fatty acids were also observed, such as the decline of ALA at the flowering stage and significant increase in 69-L2 at mature stage (**Supplementary Figure 7B**). A similar trend of variation in the fatty acid composition in PC was observed, while LA was significantly increased in 69-L1 and 69-L3 relative to HO69 at the flowering stage (**Supplementary Figure 7C**). Significant reduction in ALA was observed in the galactolipids of 69-L2 and 69-L3 at the flowering stage relative to HO69 (**Supplementary Figures 7E, G**), but not in the mature stage.

### Suppression of Target Gene Expression and Alteration in the Accumulation of Lipids and Carbohydrate in the Tubers of HO69-Derived Super-Transformation Lines

**Figure 5:**
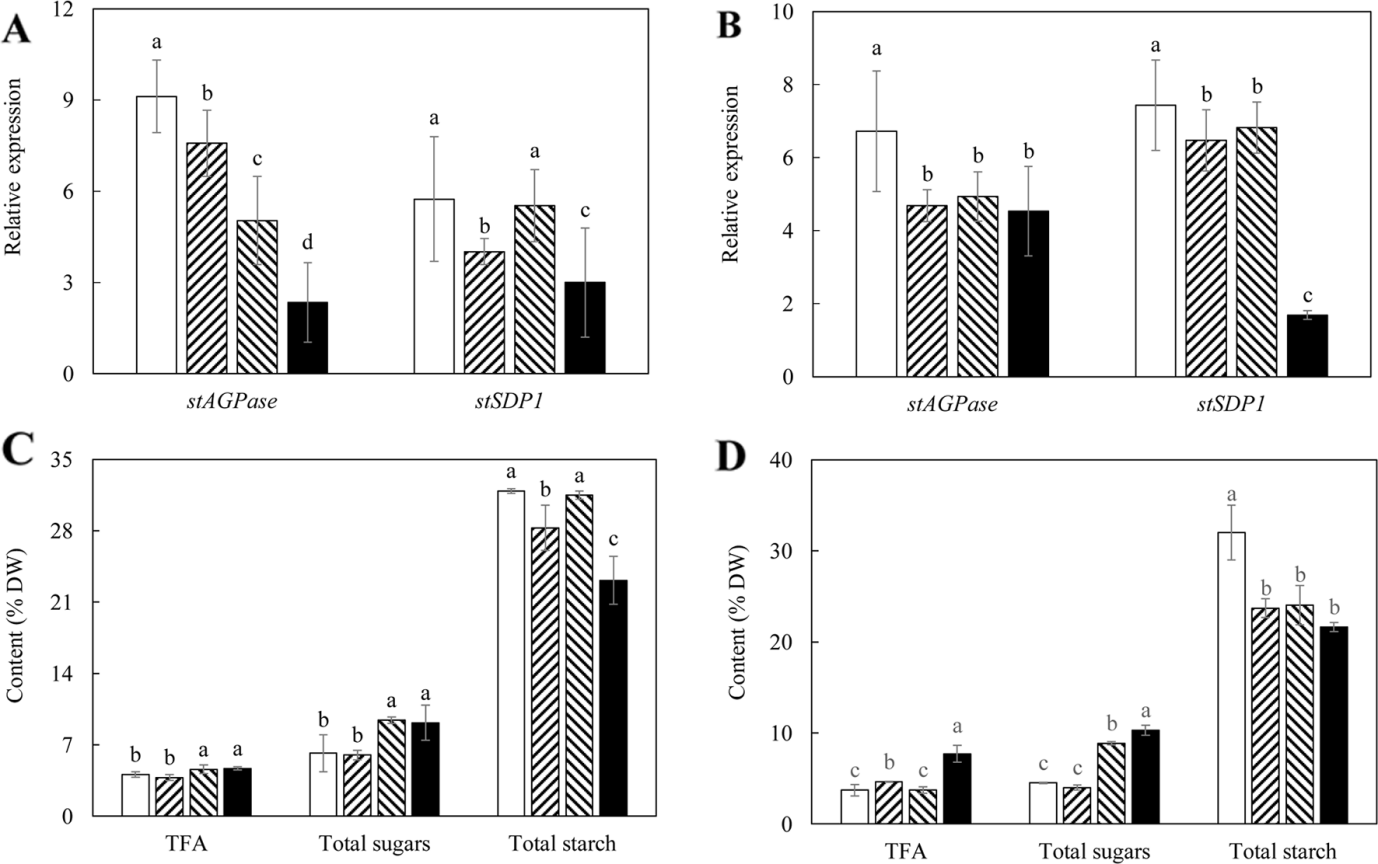
Gene expression analysis and total carbon allocation in the potato tubers of the HO69 (open bars) and three super-transformed lines, 69-L1 (bar with upward trend), 69-L2 (hatched bars) and 69-L3 (black bars) at two developmental stages. **(A)** Real-time quantitative reverse transcription polymerase chain reaction (qRT-PCR) result at the flowering stage; **(B)** Real-time qRT-PCR result at the mature stage; **(C)** Total carbon allocation at the flowering stage; **(D)** Total carbon allocation at the mature stage. The data represent the mean values ± SD of three biological replicates. Letters (a, b, c) above the bars are based on LSD, bars marked with different letters are statistically significantly different at P < 0.05.

Assessment of the expression of target genes, *StAGPase* and *StSDP1*, in the tubers of three super-transformed potato lines revealed the anticipated downregulations of both genes relative to the parent line HO69 at both the flowering and mature stages. The most severe suppression occurred in 69-L3 relative to HO69, with *StAGPase* being drastically downregulated at the flowering stage and *StSDP1* in the mature stage, while 69-L1 and 69-L2 displayed comparatively less severe reduction in expression (**Figures 5A, B**).

Corresponding to the genetic downregulation of *StAGPase* and *StSDP1* in tubers, significantly higher contents of TFA and total soluble sugars were observed in 69-L2 and 69-L3 at the flowering stage, accompanied by a reduction in starch content in 69-L1 (**Figure 5C**). At the mature stage, 69-L3 showed the biggest enhancement in TFA content up to 7.70% (DW), which was more than 2-fold increase relative to HO69 (**Figure 5D**). The accumulation of twice amount of the total soluble sugars relative to HO69 was also observed in 69-L2. All the three super-transformed lines showed significant reductions in starch contents between 21% and 24% of tuber DW from 32% in WT (**Figure 5D**).

**Figure 6:**
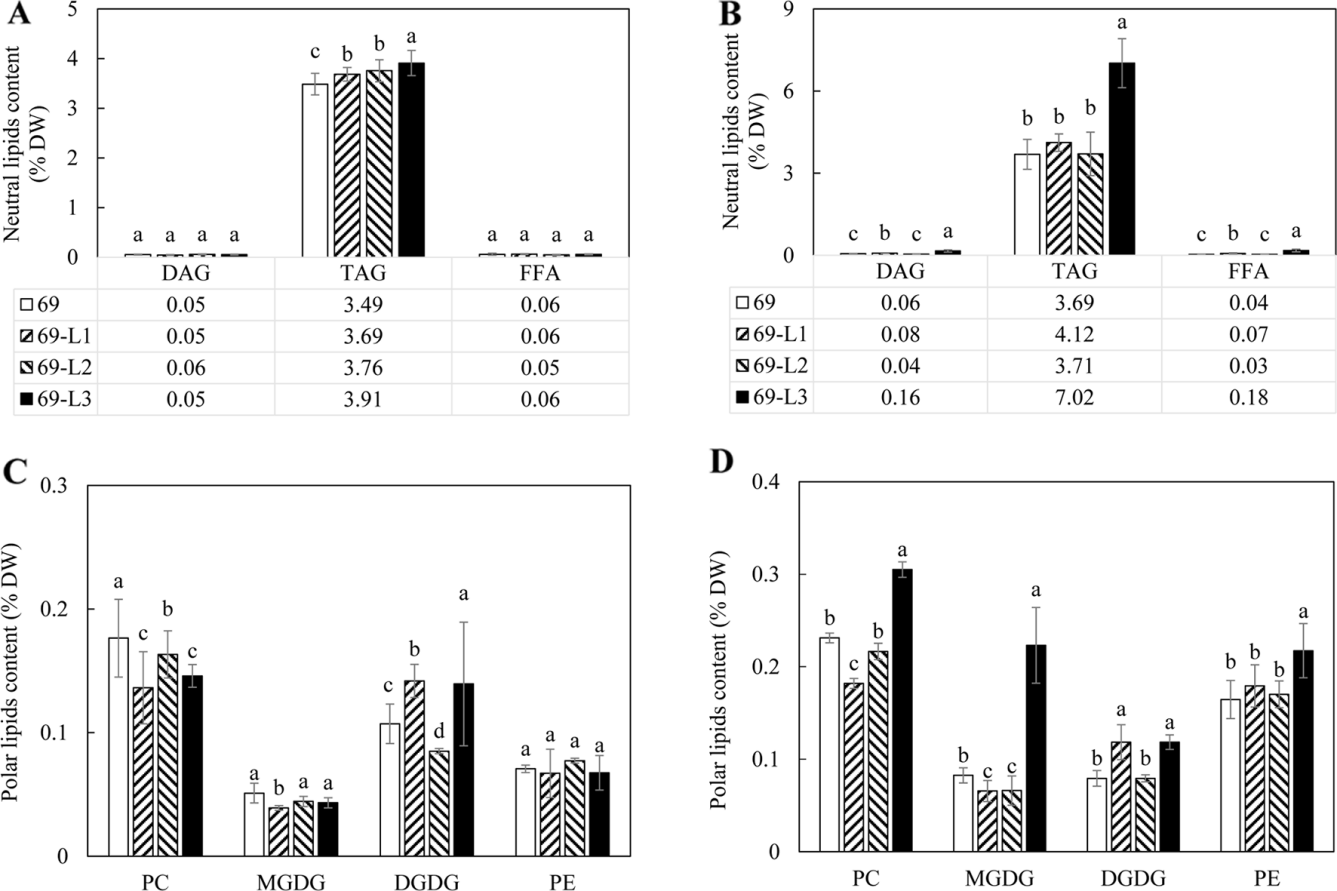
Contents of neutral and polar lipids in the potato tubers of the HO69 (open bars) and three super-transformed lines, 69-L1 (bar with upward trend), 69-L2 (hatched bars) and 69-L3 (black bars) at two developmental stages. **(A)** Neutral lipids contents at the flowering stage; **(B)** Neutral lipids contents at the mature stage; **(C)** Polar lipids contents at the flowering stage; **(D)** Polar lipids contents at the mature stage. The data represent the mean values ± SD of three biological replicates. Letters (a, b, c) above the bars are based on LSD, bars marked with different letters are statistically significantly different at P < 0.05.

A significant increase in TAG accumulation was mainly observed in 69-L3, which peaked at 7.02% at the mature stage, displaying a 2-fold increase relative to HO69 (**Figure 6B**), however not showed in the flowering stage (**Figure 6A**). Similarly, the contents of DAG and FFA were also increased, with 69-L3 showed nearly three-fold increase in DAG and four-fold increase in FFA relative to HO69. In comparison, PC contents were significantly reduced in all the three super-transformed lines compared to HO69 at the flowering stage, but DGDG was significantly increased in 69-L1 and 69-L3 (**Figure 6C**). At the mature stage, 69-L3 showed significantly enhanced accumulation of polar lipids, with MGDG being three-fold higher relative to HO69 (**Figure 6D**). Moderate yet significant variations in the amounts of polar lipids were also observed in both 69-L1 and 69-L2.

The variation of fatty acid composition in TAG of the three super-transformed lines was mainly featured by a significant reduction in oleic acid relative to HO69 at the flowering stage (**Supplementary Figure 8A**), while at the mature stage, 69-L3 showed a significant increase in oleic acid and reduction in palmitic acid (**Supplementary Figure 8B**). Moderate yet significant reduction in LA of TAG relative to HO69 was also observed in 69-L1 and 69-L2 (**Supplementary Figure 8B**). In PC, a significant increase in palmitic acid and reduction in LA relative to HO69 were observed in 69-L2 and 69-L3 at the flowering stage (**Supplementary Figure 8C**), while all the three super-transformed lines showed enhanced oleic acids relative to HO69 at the mature stage, and 69-L1 and 69-L2 displayed reduction in the LA levels (**Supplementary Figure 8D**). Relative to HO69, MGDG in these three super-transformed lines featured significantly reduced oleic acid and increased LA levels at the flowering stage (**Supplementary Figure 8E**), and a significant reduction in stearic acid at the mature stage (**Supplementary Figure 8F**). In DGDG, 69-L1 displayed significant increase in ALA, which was nearly three-fold increase compared to HO69, at the expense of both oleic acid and LA at the flowering stage (**Supplementary Figure 8G**). Similarly, almost twice as much ALA, mainly at the expense of LA, relative to HO69 was found in the DGDG of 69-L3 at the mature stage (**Supplementary Figure 8H**).

### Starch Property Analysis of the Mature Potato Tubers in the Three Super-Transformed Potato Lines

**Figure 7:**
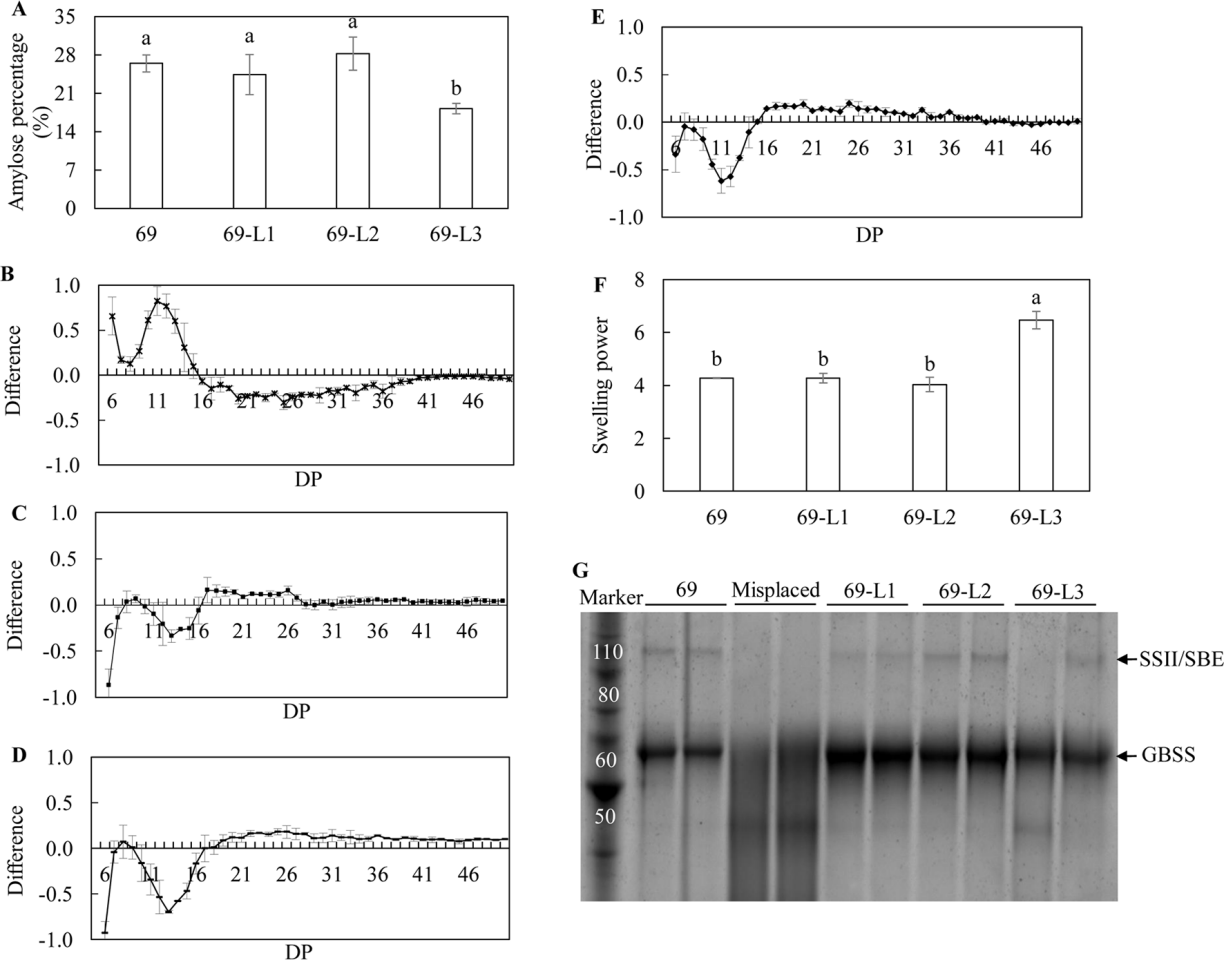
Analysis of the starch properties in the mature potato tubers. **(A)** Amylose ratio; **(B)** Difference of chain length distribution (CLD) between debranched starch of 69 and WT; **(C)** Difference of CLD between debranched starch of 69-L1 and 69; **(D)** Difference of CLD between debranched starch of 69-L2 and 69; **(E)** Difference of CLD between debranched starch of 69-L3 and 69; The CLD was reflected as DP. Each mark above the horizontal axis corresponds to the difference of a chain length in mole percentage. The error bars are standard errors. The difference value was obtained by subtracting the CLD of 69 from the three super-transformed lines, respectively. **(F)** Swelling power of potato flour; **(G)** SDS-PAGE of potato starch GBPs. The two obscure tracks between 69-L3 and 69-L2 are misplaced samples which are irrelevant with other samples. The data represent the mean values ± SD of three biological replicates. Letters (a, b, c) above the bars are based on LSD, bars marked with different letters are statistically significantly different at P < 0.05.

Among the three super-transformed lines, 69-L3 had a significant reduction in amylose content, which was 18.19%, nearly 1.5-fold lower than that in HO69 (**Figure 7A**). CLD analysis revealed that the parent line HO69 with enhanced TAG accumulation, compared to the WT potato, displayed a preference to short chains (DP <11) rather than intermediate chains between DP12 and DP19 (**Figure 7B**). However, in the debranched starch of the three super-transformed lines, significantly reduced formation of short chains was observed in 69-L2 and 69-L3 compared to HO69 (**Figures 7D, E**), and to a lesser extent in 69-L1 but not statistically significant (**Figure 7C**). Meanwhile the accumulation of intermediate chains was significantly reduced in 69-L2 which also showed significant increase in long chains (DP >19) relative to HO69 (**Supplementary Table 2**). Only 69-L3 displayed significantly enhanced swelling power of the tuber flour compared to HO69, which was not identified in the other two lines (**Figure 7F**). The SDS-PAGE analysis of GBPs showed visually enhanced accumulation of GBSS protein in 69-L1 and 69-L2 relative to HO69, but relatively decreased in 69-L3. The protein bands representing the SSII/SBE showed a decreased abundance in 69-L3 relative to HO69 (**Figure 7G**), analogous to the analysis made in the digitalization of protein bands intensity, of which 69-L3 showed almost 0.9-fold drop in both GBSS and SSII/SBE (**Supplementary Table 1**).

### Microscopic Analysis of Lipid Droplets and Starch Granules in Mature Transgenic Potato Tubers

**Figure 8:**
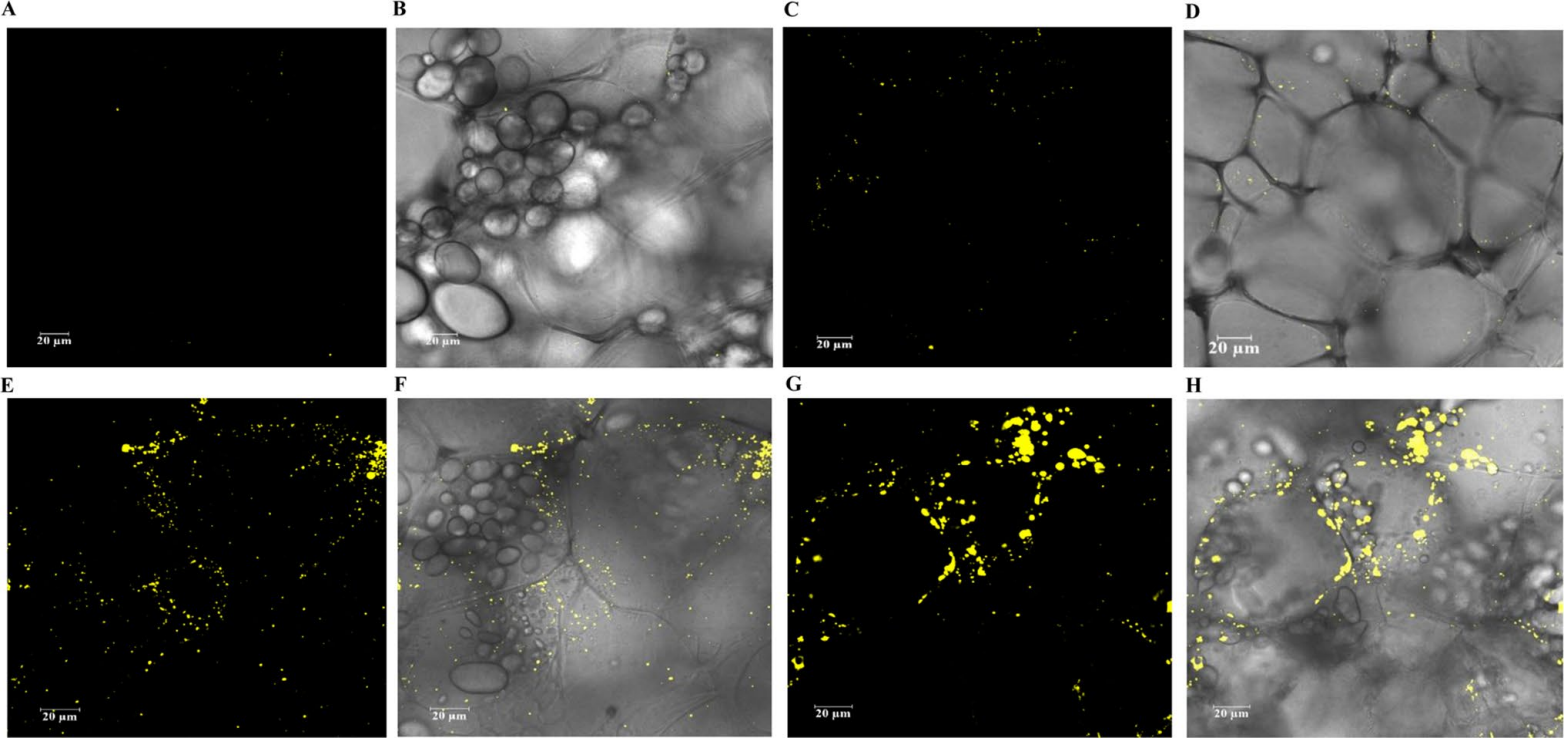
Confocal scanning microscopy analysis of the LDs distribution in mature potato tubers. The LDs were stained with BODIPY (yellow), bright-field images of the unstained starch granules and cell wall structures formed the major background contrasted to the LDs. **(A**, **B)** Visualization of LDs in WT. **(C**, **D)** Visualization of LDs in WT-L5. **(E**, **F)** Visualization of LDs in HO69. **(G**, **H)** Visualization of LDs in 69-L3. The scale bars are located in the lower left corner for each photograph, sizes of the scale bars are all normalized to 20 µm.

The cellular distribution of LDs in the mature potato tubers was visualized with confocal scanning microscopy. Two transgenic lines, WT-L5 and 69-L3, which displayed the most significant alterations in the contents of TAG and starch were selected for comparison with WT and HO69. Both round and oval shaped LDs were observed following the staining with BODIPY, with unstained starch granules in the background. Compared to WT, which displayed highly abundant starch granules and rather limited numbers of LDs (**Figures 8A, B**), enhanced accumulation of LDs was displayed in WT-L5 but accompanied with apparent reduction in the abundance and size of starch granules (**Figures 8C, D**). In the high oil parent line HO69, abundant LDs were observed and broadly distributed in the tuber section, with starch granules of variable sizes distributed within the cell compartments (**Figures 8E, F**). Such observations on HO69 was consistent with our previous study (Liu et al., 2017). By comparison, the super-transformed line 69-L3, appeared to have enlarged LDs and reduced number of small LDs compared to HO69 (**Figure 8G**). Relatively, fewer starch granules than in HO69 were also evident in 69-L3 (**Figure 8H**).

**Figure 9:**
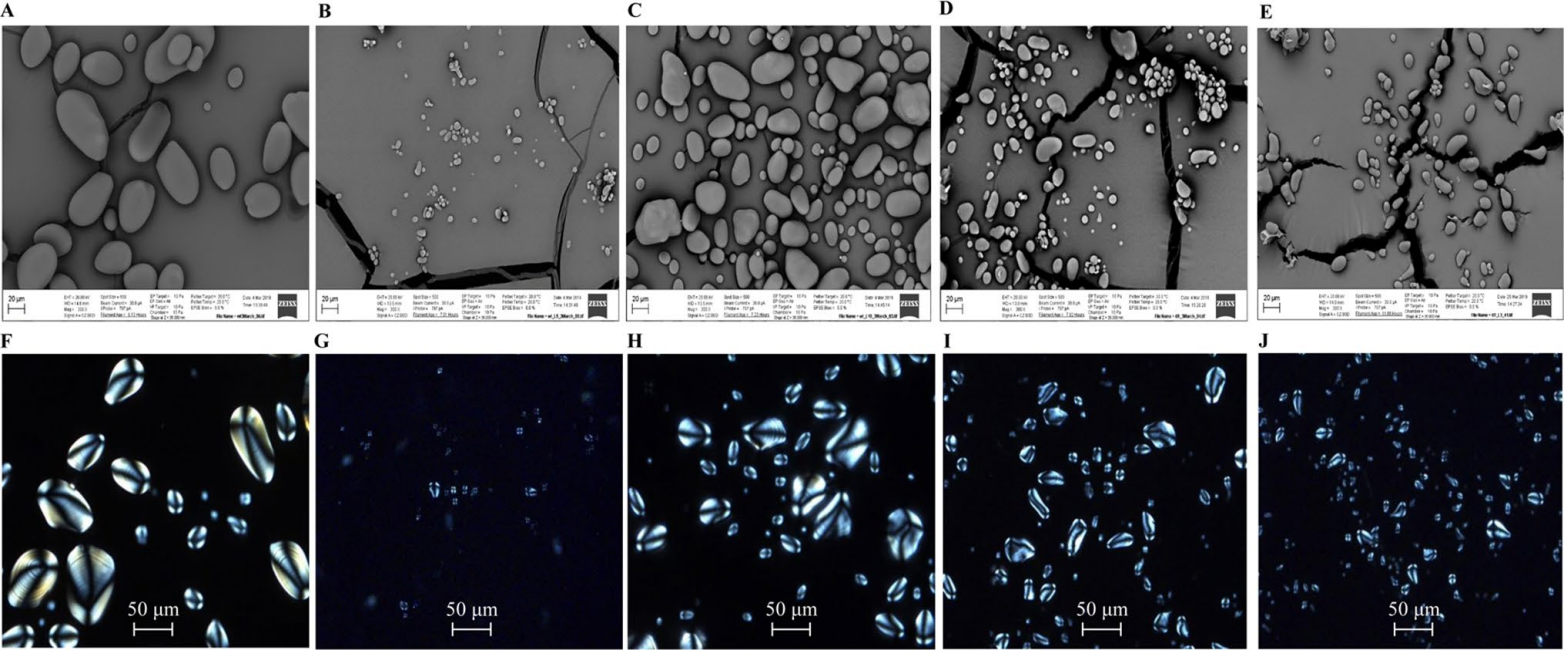
Microscopic observation of starch granules in mature potato tubers. **(A)** scanning electron microscopy (SEM) image of WT; **(B)** SEM image of WT-L5; **(C)** SEM image of WT-L10; **(D)** SEM image of HO69; **(E)** SEM image of 69-L3; **(F**) Polarized light image of WT; **(G)** Polarized light image of WT-L5; **(H)** Polarized light image of WT-L10; **(I)** Polarized light image of HO69; **(J)** Polarized light image of 69-L3. The starch granules were observed under a view of 300× magnification in the SEM, with scale bars located in the lower left corner for each photograph. Sizes of the scale bars are all normalized to 20 µm. For the images taken by light microscopy, the scale bars are located in the lower middle position of each photograph. Size of the scale bars are normalized to 50 µm.

Morphological variation of starch granules in the mature tubers was analyzed with the scanning electron microscopy (SEM). Regular and evenly distributed starch granules in WT were found, about 20–60 µm in length and in an oval or spherical shape with a smooth surface (**Figure 9A**). However, in WT-L5, the size and number of the starch granules were drastically reduced, many of them with an irregular ellipsoid shape (**Figure 9B**). The starch granules in WT-L5 showed a nearly 10-fold reduction in the size (< 2 µm) when compared to WT, while the largest was around 10 µm. In contrast, the alteration in size and shape of starch granules in WT-L10 was relatively moderate but still apparent relative to WT (**Figure 9C**). Compared to HO69 (**Figure 9C**), 69-L3 did not show any evident morphological differences but had a declining trend in starch granule size (**Figure 9D**). The observation through light microscopy revealed that the birefringence of starch granules, featured by the Maltese cross and brightness, remained almost unaltered between the transgenic potatoes and their parent lines under the polarized light. Specifically, the crystalline order of the starch granules between WT (**Figure 9F**) and the two selected WT-derived transgenic lines (**Figure 9G–H**) did not appear to be affected, even though WT-L5 showed significant reduction in starch granule size (**Figure 9E**). There was no discernible difference between HO69 and the super-transformed line 69-L3 on the starch crystalline order either (**Figures 9I, J**).

## Discussion

In this study, we have shown that the simultaneous downregulation of *StAGPase* and *StSDP1* induced significant reconstitution in the carbon partitioning in potato vegetative tissues. This was concluded from the transgenic potato plants with a duplex RNAi construct specifically targeting the expression of *AGPase* and *SDP1* genes, which displayed significant alteration in the composition of lipids, total soluble sugars and starch, representing a coordinated carbon redistribution in the transgenic potatoes. This is consistent with the previous studies on high oil potatoes (Hofvander et al., 2016; Liu et al., 2017). Variation in the carbon metabolism of the two WT-derived transgenic lines selected for this study may represent the difference in the effectiveness of RNAi downregulation of the two selected target genes. However, in the HO69-derived super-transformation, due to the low-regeneration rate of transgenic potato plants, only three lines were obtained, with 69-L3 displaying the most distinct RNAi phenotype. Therefore, all of the three super-transformed lines were used for further analysis.

Potato mainly propagates through tuber-cutting, a form of vegetative propagation, which does not facilitate transgene stacking by sexual crossing. The selected HO69 was hence super-transformed with the duplex RNAi construct through the Agrobacterium-mediated gene transformation approach. Because the kanamycin resistance conferred by neomycin phosphotransferase II (*NPTII*) had already been used as the selectable maker in the generation of HO69 (Liu et al., 2017), hygromycin was used as an alternative selectable agent in the super-transformation. The use of *HPH* as the selectable marker is clearly less effective than *NPTII* in our experiment as the hygromycin could impose some level of stress on the explants (Barrell et al., 2002; Sundar and Sakthivel, 2008), which may explain the relatively lower frequency of transgenic plants compared with our previous study (Liu et al., 2017). However, we could not rule out the possibility that the stacking of *StAGPase-StSDP1* RNAi cassettes with the original three transgenes, *AtWRI1-AtDGAT1-siOLEOSIN* contained in the pJP3506 vector (Liu et al., 2017) may also have had some potential repercussions on the transformed cells in terms of metabolism (Halpin, 2005; Baltes and Voytas, 2015), thereby severely restricting the number of transgenic plants coming through the plant regeneration system.

In the leaves of the selected transgenic lines, WT-L10 and 69-L3 displayed enhanced lipid biosynthesis, whereas other lines showed alteration in the accumulation of carbohydrates at variable levels. In the tubers, the two selected WT-derived lines, WT-L5 and WT-L10, as well as the super-transformed line 69-L3 all showed enhanced lipid production. This may be largely due to the variable RNAi effects of the two target genes. As a direct result of the carbon reallocation following *StAGPase* suppression, it is evident that more carbon molecules released from the starch anabolism would be diverted into other carbon metabolic pathways (Sweetlove et al., 1998). AGPase is the rate-limiting enzyme exclusively catalyzing the production of ADP-glucose, which is the direct substrate for starch biosynthesis, thus it is also considered the “valve” controlling the carbon flux distribution from glycolysis (Barıs et al., 2009; Lee et al., 2009). Downregulation of *AGPase* expression in plant would inevitably lead to the re-distribution of carbon flux, which may have potentially provided more available carbon donors for the lipid biosynthesis and compartmentalization (Sanjaya et al., 2011; Yu et al., 2018), as well as the accumulation of soluble sugars (Muller-Rober et al., 1992; Geigenberger 2003; Klaus et al., 2004).

Meanwhile, the suppression of *StSDP1* may have prevented the rapid turnover of TAG and enabled their accumulation in the form of LDs (Eastmond, 2006; Walther and Farese, 2012; D’Andrea, 2016). Kelly et al. (2013) reported that the individual disruption of *SDP1* led to a substantial and continuous accumulation of TAG in the root and stem tissues of Arabidopsis during plant development, and to a lesser extent in leaf. Similarly in the transgenic potato leaves, the enhancement of TAG biosynthesis was observed, but not in WT-L5. This may be due to the difference in RNAi effects, the amount of soluble sugars was increased in WT-L5 leaf as a result of the carbon reallocation from starch decrease *via* the downregulation of *StAGPase*. However, the simultaneous suppression of *StSDP1* did not effectively contribute to TAG maintenance (**Figure 2C**). But the TAG abundance, particularly in the mature tubers of 69-L3, which was doubled to 7.02% of tuber DW relative to parent line HO69, could be the direct result of downregulating *StSDP1*, which is consistent with the study in tobacco (Vanhercke et al., 2017). The enhancement of PC and PE in the mature tubers of WT-L5 (**Figures 3C, D**) and 69-L3 (**Figure 6D**) could be a side effect of the *StSDP1* suppression. As recent study has revealed that *SDP1* plays a key role in TAG turnover through being timely delivered *via* the peroxisomal extension to disintegrate LDs from the surrounding phospholipid membranes (Thazar-Poulot et al., 2015). Through the inhibition in the activity of *SDP1*, the packaging of LDs *via* the phospholipid monolayer might be indirectly re-enforced (Matos and Pham-Thi, 2009). By contrast, the increase of galactolipids in transgenic potato vegetative tissues, to a certain extent, may be owing to the expanded carbon provision for the *de novo* plastidial fatty acid biosynthesis after repressing starch production (Li et al., 2010; Yu et al., 2013; Takahashi et al., 2018).

Nevertheless, in the transgenic potatoes, for both WT and the high oil genetic background, the TAG biosynthetic pathway may not have been able to utilize all the extra carbon derived from the repression of starch production and TAG turnover, which may have also resulted in the increased accumulation of soluble sugars. Although we could not rule out the possibility that the enhancement of sugars and TAG productions may be two independent metabolic events as a result of the downregulation of *StAGPase* and *StSDP1* in potatoes, the significant increase in lipid biosynthesis, which are the byproducts of carbohydrate metabolism in potato tubers suggests that the carbon allocations between lipids and carbohydrate are highly orchestrated in potato non-photosynthetic tissues. Likewise, the carbon re-allocation in the transgenic potato leaves also showed the same tendency, particularly in WT-L10 and 69-L3, which was generally consistent with the study in the Arabidopsis mutant deficient in starch biosynthesis, leading to the enhanced lipid biosynthesis and turnover (Yu et al., 2018). Consequently, it was hypothesized that the *CaMV-35S* promoter induced constitutive RNAi silencing of *StAGPase* may be the dominant factor for the overall carbon partitioning variation in transgenic potato plants, including both the increased sugar levels and fatty acid biosynthesis, while the accumulation of TAG, and phospholipids to a lesser extent, may be mainly due to the downregulation of *StSDP1*. Similar results were also observed in the mutagenesis of the *SSIIa* gene in barley, which encodes one of the major starch biosynthetic enzymes localized on the downstream of AGPase. The starch content in the barley mutant was significantly reduced relative to WT, while lipid and sugar contents were increased to 1.7 and 8.6-fold, respectively (Clarke et al., 2008; Li et al., 2011). However, the differentiated RNAi effects on transgenic potato leaves and tubers, as well as the discrepancy for the discriminatory synthesis of sugars and lipids, still warrant further investigation (Tiessen and Padilla-Chacon, 2013; Zhai et al., 2017; Zhai et al., 2018).

The fatty acid composition in TAG of both leaf and tuber of the two selected WT-derived lines showed significantly increased ALA but decreased LA levels relative to WT at the mature stage, which was consistent with the study in Arabidopsis (Fan et al., 2014). In the super-transformed potato lines, the fatty acid composition was different. Consistent with the high oil transgenic tobacco leaf with the downregulation of *SDP1* expression (Vanhercke et al., 2017), drastic reduction in ALA was observed in the leaves of 69-L2 and 69-L3 at the flowering stage, as well as in PC and galactolipids, but not in tubers. To a certain degree, the variations in fatty acid composition among species and tissues at disparate physiological stages may be due to the differential acyl flux regulation (Ohlrogge and Jaworski, 1997; Slocombe et al., 2009; Bates et al., 2009; Bates et al., 2016; Marchive et al., 2014). The synergistic manipulation of *AtWRI1-AtDGAT1-SiOLEOSIN* expressions have induced a hallmarked fatty acid distribution as enhanced LA at the expense of ALA in high oil transgenic plants (Vanhercke et al., 2014; Liu et al., 2017). When the *SDP1* retromer complex mediated LD-peroxisome communication during the TAG turnover (Thazar-Poulot et al., 2015) was RNAi suppressed, the fatty acid mobilizations in the HO69-derived super-transformed potato lines may be further impacted, leading to more diversified fatty acid distributions in vegetative tissues.

Starch composition and structure were significantly altered in the mature transgenic potato tubers relative to their parent lines. The decreased amylose content, varied amylopectin CLD and accumulation of GBPs suggested the activity of starch biosynthetic enzymes have been affected in the transgenic potatoes derived from both the WT and high oil genetic backgrounds (Luo et al., 2015), leading to altered starch functionality as indicated by the differed swelling power values (Jane et al., 1999; Srichuwong et al., 2005; Vamadevan and Bertoft, 2015). Amylose is the linear long chain polysaccharide molecule with few branches associated inside the starch granule, the biosynthesis of amylose is directly controlled by the GBSS enzyme, which is also one of the GBPs (Denyer et al., 2001; Vamadevan and Bertoft, 2009). However, the increase of GBSS protein in the transgenic potatoes relative to their parent lines was not consistent with the reduction of amylose content. This suggests the GBSS protein accumulation in transgenic potato starch may be a pleiotropic effect of downregulating *AGPase*, while the altered CLD may be due to the reduction in SSII and SBE proteins. We propose that the suppression of *StAGPase* following variable RNAi effects among different transgenic potato plants may be the dominant factor leading to all these differences, because a limited ADP-glucose provision may cause the substrate competition in starch biosynthesis (Lloyd et al., 1999). The negative feedback regulation on the gene expression involved in the sequential biosynthesis of amylose and amylopectin, as the result of *AGPase* downregulation, could induce the alteration of the amylopectin chain length and branch frequency in the starch granule formation thereafter (Kuipers et al., 1994; Lloyd et al., 1999; Luo et al., 2015). Further, with the reduction of amylose, the proportion of amylopectin may increase, leading to enhanced swelling power in WT-L10 and 69-L3. However, similar impacts were not reflected in WT-L5, which may be partially due to the slight decline in amylopectin chain length between DP12 and DP16 (**Figure 4B**). Such a result was similar with the *SSIIa* mutation in wheat (Konik-Rose et al., 2007).

As further revealed in the microscopic analysis, the LD and starch granule derived from the selected transgenic potato tubers both displayed consistent variation with the biochemical results. Obviously, the changes in the starch composition and the starch granule morphology were indicative for the interactions between *AGPase* and other starch biosynthetic enzymes. Additionally, the significantly increased LDs distribution in WT-L5, and the enlarged size of LDs in 69-L3, might be caused by the fusion of small LDs (Fan et al., 2014; Vanhercke et al., 2017). Although the enhancement of LD formation could be largely owing to the *StSDP1* downregulation, and the suppression of *StAGPase* induced alteration in starch granule morphology, the microscopic results still implicated an underlying correspondence between the LD biogenesis and starch biosynthesis in potato tubers. A further comparison with the individual downregulation of *StAGPase* and *StSDP1*, respectively, in both the WT and high oil genetic potato backgrounds may bring more insights in this respect.

Previous efforts in downregulating *AGPase* alone in potato tubers resulted in the reduction of starch, without increasing the lipid content (Klaus et al., 2004). We demonstrated in this study that the carbon reallocation from starch to fatty acids and lipids may be possible when *StAGPase* and *StSDP1* were simultaneously suppressed. Not only were the contents of starch, lipids, and soluble sugars, but also the fatty acid composition, starch granule properties were significantly altered. The expression of *AtWRI1* transcriptional factor in the HO69-derived super-transformation may be a potential factor limiting the valid RNAi suppression of *StSDP1*, and the “feedback inhibition relationship” between the TAG lipase and LD integral protein *OLEOSIN* may be another one (Vanhercke et al., 2017; Huang 2018). Unlike genome editing, RNAi occurs on the post-transcriptional level (Jagtap et al., 2011; Watanabe, 2011), its effects are largely dependent on the insertion site of the RNAi cassettes into potato genome, which are somewhat unpredictable. Moreover, the significantly enhanced production of membrane lipids, particularly MGDG and DGDG, in the transgenic tubers of WT-L5 and 69-L3, may suggest a possibly reinforced fatty acid and/or lipid biosynthesis within the non-photosynthetic plastid, the amyloplast in potato, which was consistent with our previous report (Liu et al., 2017). Amyloplast usually plays the major role in starch biosynthesis, only to a lesser extent in the rather limited fatty acids and plastidial lipid production in potato (Ferreira et al., 2010; Nazarian-Firouzabadi and Visser, 2017). By comparison, the galactolipids abundantly synthesized in chloroplast are not only actively involved in the maintenance of the cellular homeostasis of green tissues (Chapman et al., 2013; Xu and Shanklin, 2016), but also the responsive machinery tackling various plant abiotic/biotic stresses (Welti et al., 2002; Moellering et al, 2010). In our study, the manipulations towards the endosperm reticulum (ER)-specific Kennedy pathway have induced substantial accumulation of plastidial galactolipids in tubers. One hypothesis is that the tuber specific B33 promoter controlled expression of *AtWRI1* transcriptional factor, as a critical regulatory gene localized on the upstream of the global lipid metabolic networks (Ma et al., 2013; Ma et al., 2016), may have contributed mostly to the excessive production of *de novo* fatty acids as source for the prokaryotic galactolipid production in potato amyloplast (Liu et al., 2017), which might be also enhanced by the RNAi silencing of *StAGPase* to donate more carbon. However, questions such as how the expression of transgenes affect the amyloplast biogenesis (*e.g.* structure and functionality) in high oil potato tubers, how the transgenes contribute to the plastidial lipids biosynthesis, as well as the interactions among the plastid- and ER-specific genes during the carbon partitioning still remain to be further explored. High throughput characterization methods such as transcriptomics, lipidomics, or metabolomics would be useful to better understand the mechanisms underlying these distinct transgenic phenotypes, while high-resolution microscopic technologies like transmission electron microscopy (TEM) could also be applied to depict the structural dynamics of the intracellular organelles in potato tuber. Further, the overproduction of sugars implies a yet limited enhancement of TAG biosynthesis in the transgenic potatoes. There may still be potential to more efficiently direct the carbon flux into the oil production in potato, for example *via* changes to sucrose metabolism (Vanhercke et al., 2019). The results embodied in this study warrant further exploration of the lipid and carbohydrate interactions in plant non-seed biomass tissues. Also, other important storage components such as protein and fiber need to be taken into account in the metabolic engineering of valuable co-products from plants.

## Experimental Procedures

### Concurrent Gene Silencing of StAGPase and StSDP1 Through RNAi

Selected DNA fragments derived from *StAGPase* and *StSDP1* genes were fused and constructed into an inverted repeat configuration, which was placed behind the *CaMV-35S* promoter in the pWBVec2 vector (Wang et al., 1998). For amplifying the cDNA fragments for *StAGPase* and *StSDP1* genes, total RNAs were isolated from mid-development potato tubers using RNeasy Mini Kit (QIAGEN, Hilden, Germany). The first strand cDNA was synthesized by reverse transcription using Superscript III RT/Platinium Taq High fidelity Enzyme Mix kit (Life Technologies, Calsbad, CA) with the following primers which specifically amplified a 500 bp fragment of the two target genes, *StAGPase* sense: 5’-ACAGACATGTCTAGACCCAGATG-3’, *StAGPase* antisense: 5’-CACTCTCATCCCAAGTGAAGTTGC-3’; *StSDP1* sense: 5’- CTGAGATGGAAGTGAAGCACAGATG-3’, *StSDP1* antisense: 5’-CCATTGTTAGTCCTTTCAGTC-3’. The PCR products were then purified by gel fractionation and ligated into the pGEMT Easy vector (Promega, Madison, WI) which was then transformed into *E. coli* cells for selecting target colonies. After the DNA sequencing verification, the two fragments representing *StAGPase* and *StSDP1* genes, respectively, were fused by the overlapping PCR (Bryksin and Matsumura, 2010), then incorporated into the pKannibal vector to clone the sense and antisense fragments. Restriction sites *Bam*HI and *Hin*dIII were selected for cloning in one orientation and *Kpn*I and *Xho*I for the antisense orientation. Subsequently, the resulting RNAi structure, together with the CaMV-35S promoter and OCS terminator sequence were released from the *Not*I site of pKannibal vector and cloned into a binary vector pWBVec2 with hygromycin as plant selectable marker. This binary vector was further introduced into *A. tumefaciens* AGL1 strain through electroporation for potato transformation. The configuration map the pWBVec2 construct was illustrated in **Supplementary Figure 9**.

### Potato Transformation

Aseptic seedlings of the HO69 (Liu et al., 2017) were prepared before tissue culture transformation. The establishment of this donor plant system used the method described by Mohapatra and Batra (2017) (**Supplementary Figure 10**). Both the WT- and super- transformations with the same RNAi construct were carried out as described in Liu et al. (2017), except that sucrose concentration used in the super-transformation was reduced to 15 g/L in all the MS media throughout the entire transformation process. This modification in protocol was to address the sensitivity to sucrose of explants derived from HO69 due to *WRI1* overexpression (Cernac and Benning, 2004). The obtained T_0_ plants were maintained in the phytotron glasshouse (24/20°C, 16 h photoperiod) at CSIRO Black Mountain Science and Innovation Park, Canberra. After the molecular verification and selection, several lines with representative transgenic traits were selected to synchronically propagate with WT and HO69 under the same conditions for further characterization. Two developmental stages, the flowering stage (>70% of flowers in one plant are blossoming and developing tubers are visible) and the mature stage (>50% aging leaves and >80% mature tubers are observable) were analyzed.

### Genetic Verification of Transgenic Plants and qRT-PCR Analysis of Gene Expressions

The Phire^™^ Plant Direct PCR Kit (Thermo Fisher Scientific, Waltham, MA) was applied to quickly verify the presence of selectable marker gene hygromycin-B-phosphotransferase (*HPH*) in the DNAs isolated from the leaves and tubers of T_0_ potato plants. Primers used for the amplification of a 200 bp region of the hygromycin gene were: sense 5’-GACCTGCCTGAAACCGAACT-3’; antisense 5’-TCGTCCATCACAGTTTGCC-3’. The PCR reaction program was as follows: initial denaturation at 95 °C for 3 min, followed with 40 cycles of 95 °C for 10 s, 60 °C for 30 s, 72 °C for 30 s. The PCR products were visualized on 1% agarose gel to initially verify the transgenic plants.

Total RNA from healthy and fully expanded leaves of WT, HO69, and the selected transgenic potato lines were isolated using the RNeasy Mini Kit (Qiagen) as specified in the manufacture’s protocol, and the quantity was measured using a Nanodrop spectrophotometer ND 1000 (Thermo Fisher Scientific). The total RNA from mature potato tubers were extracted by the cetyl trimethyl ammonium bromide (CTAB) method (Reynolds et al., 2019) and purified using a RNeasy MinElute Cleanup Kit (Qiagen) following manufacturer’s instructions. Real-time quantitative reverse transcription polymerase chain reaction (qRT-PCR) was applied to study the gene expression of *StAGPase* and *StSDP1* in potato vegetative tissues, with the *S. tuberosum CYCLOPHILIN (stCYP)* gene as the reference gene. The targeting primers for the *StCYP* gene are sense: 5’-CTCTTCGCCGATACCACTC-3’; antisense: 5’-CACACGGTGGAAGGTTGAG-3’. Primers for *StAGPase* are sense: 5’-CACACAATTCAACTCTGCCTC-3’; antisense: 5’-GCTCCTCAAACAACCACAG-3’, and primers for *StSDP1* are sense: 5’-GTTGTCACTCGTGGACTCG-3’; antisense: 5’-CTTGGACAAGATCAGGTGG-3’. The real-time qRT-PCR reaction was developed in a Biorad 96 well PCR machine (BioRad, Hercules, CA) programmed as 95°C for 3 min, 39 cycles of 95°C for 10 s, 56°C for 30 s, and 72°C for 30 s, with the FastStart Universal SYBR Green Master (ROX) kit (Roche, Indianapolis, IN) as the supporting reaction system. Gene expression was calculated by following the 2^-ΔΔCt^ method (Livak and Schmittgen, 2001).

### Lipid and Carbohydrate Biochemical Analysis

Healthy potato fully expanded leaves and tubers were freshly sampled at the two developmental stages, and immediately freeze-dried for 72 h. The lipid analysis including TFA, FFA, neutral lipids (*i.e.* TAG, DAG), and polar lipids (*i.e.* PC, MGDG, DGDG, PE and PG) were developed as previously described by Vanhercke et al. (2018) through the TLC and GC analysis and carbohydrates analysis including total starch and total soluble sugars were measured as described by Liu et al. (2017) *via* the Megazyme Total Starch Kit (Megazyme International Ireland, Bray, Ireland) following the manufacturer’s instruction.

### Starch Property Analysis of Mature Potato Tubers

Potato starch was isolated from mature and healthy tubers sampled at the mature stage as described in Liu et al. (2017). Estimation of the amylose content was made by following the coloration method described in Morrison and Laignelet (1983). The CLD analysis was carried out using the capillary electrophoresis (CE), following starch debranching as described in Luo et al. (2015). The SDS-PAGE analysis of GBPs, with starch content used to standardize the amount was carried out following the method described by Luo et al. (2015). The digital analysis of protein bands intensity on SDS gels was developed on the ImageScanner III machine (GE Healthcare Life Science) via the Totallab Quant Software (Totallab, Newcastle, USA) by following manufacturers’ instructions. The swelling power analysis of potato tuber flour was carried out following Konik-Rose et al. (2007).

### Microscopic Analysis

Confocal scanning microscopy analysis of LDs was carried out as previously described (Vanhercke et al., 2018). SEM and light microscopy analyses of the potato tuber starch granules were carried out as previously described (Liu et al., 2017).

### Statistical Analysis

GenStat 9.0 software was used to calculate the least significant difference (LSD) value of all data for multiple comparisons.

## Data Availability Statement

The datasets generated for this study are available on request to the corresponding author.

## Author Contributions

XX designed the research, performed the experiments and wrote the paper. TV, PS, and SS provided precious guidance all along the research as project supervisors. JL and CK-R assisted the starch experiments. SA and DH assisted the preparation of the transgenic construct. LV assisted the confocal microscopic analysis. LT assisted the glasshouse maintenance. PJS, ZL and QL conceived and designed the project, and improved the manuscript.

## Funding

This project is supported by CSIRO Agriculture and Food and The University of Sydney.

## Conflict of Interest

The authors declare that the research was conducted in the absence of any commercial or financial relationships that could be construed as a potential conflict of interest.
