# Supplementary material for: Upregulated Lipid Biosynthesis at the Expense of Starch Production in Potato (Solanum tuberosum) Vegetative Tissues via Simultaneous Downregulation of ADP-Glucose Pyrophosphorylase and Sugar Dependent1 Expressions
Source: Front Plant Sci. 2019 Nov 12;10:1444. doi: 10.3389/fpls.2019.01444 (PMC6861213; doi:10.3389/fpls.2019.01444)
Supplement: Supplementary file 1 [file DataSheet_1.docx]

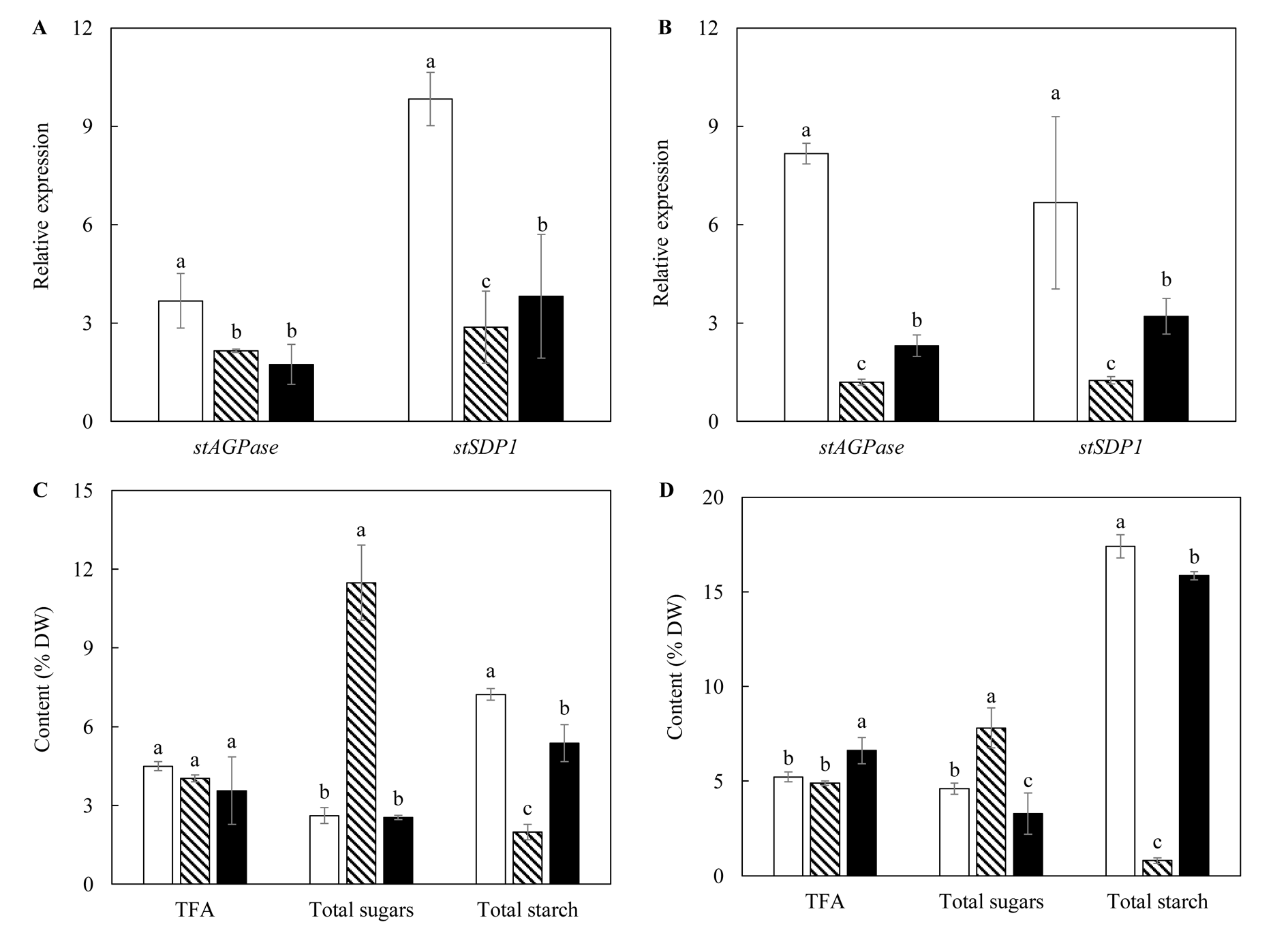


**Supplementary Figure 1.** Gene expression analysis and total carbon allocation in the potato leaves of WT (open bars) and the two selected WT-derived lines, WT-L5 (hatched bars) and WT-L10 (black bars) at two developmental stages. (A) Real-time qRT-PCR result at the flowering stage; (B) Real-time qRT-PCR result at the mature stage; (C) Total carbon allocation at the flowering stage; (D) Total carbon allocation at the mature stage. The data represent the mean values ± SD of three biological replicates. Letters (a, b, c) above the bars are based on LSD, bars marked with different letters are statistically significantly different at P<0.05.


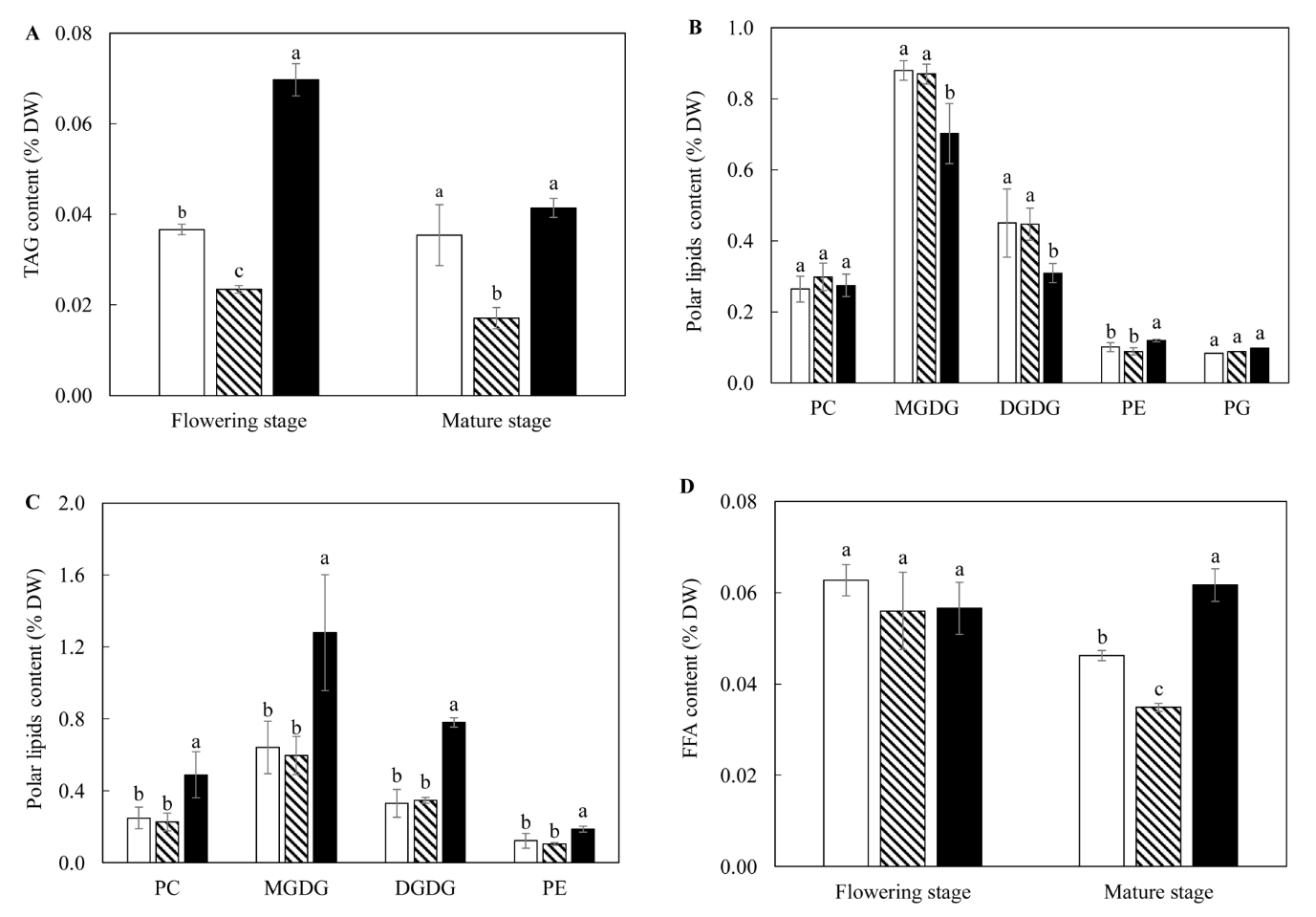


**Supplementary Figure 2.** Contents of TAG, polar lipids and FFA in the potato leaves of WT (open bars) and the two selected WT-derived lines, WT-L5 (hatched bars) and WT-L10 (black bars) at two developmental stages. (A) TAG contents; (B) Polar lipids contents at the flowering stage; (C) Polar lipids contents at the mature stage; (D) FFA contents. The data represent the mean values ± SD of three biological replicates. Letters (a, b, c) above the bars are based on LSD, bars marked with different letters are statistically significantly different at P<0.05.


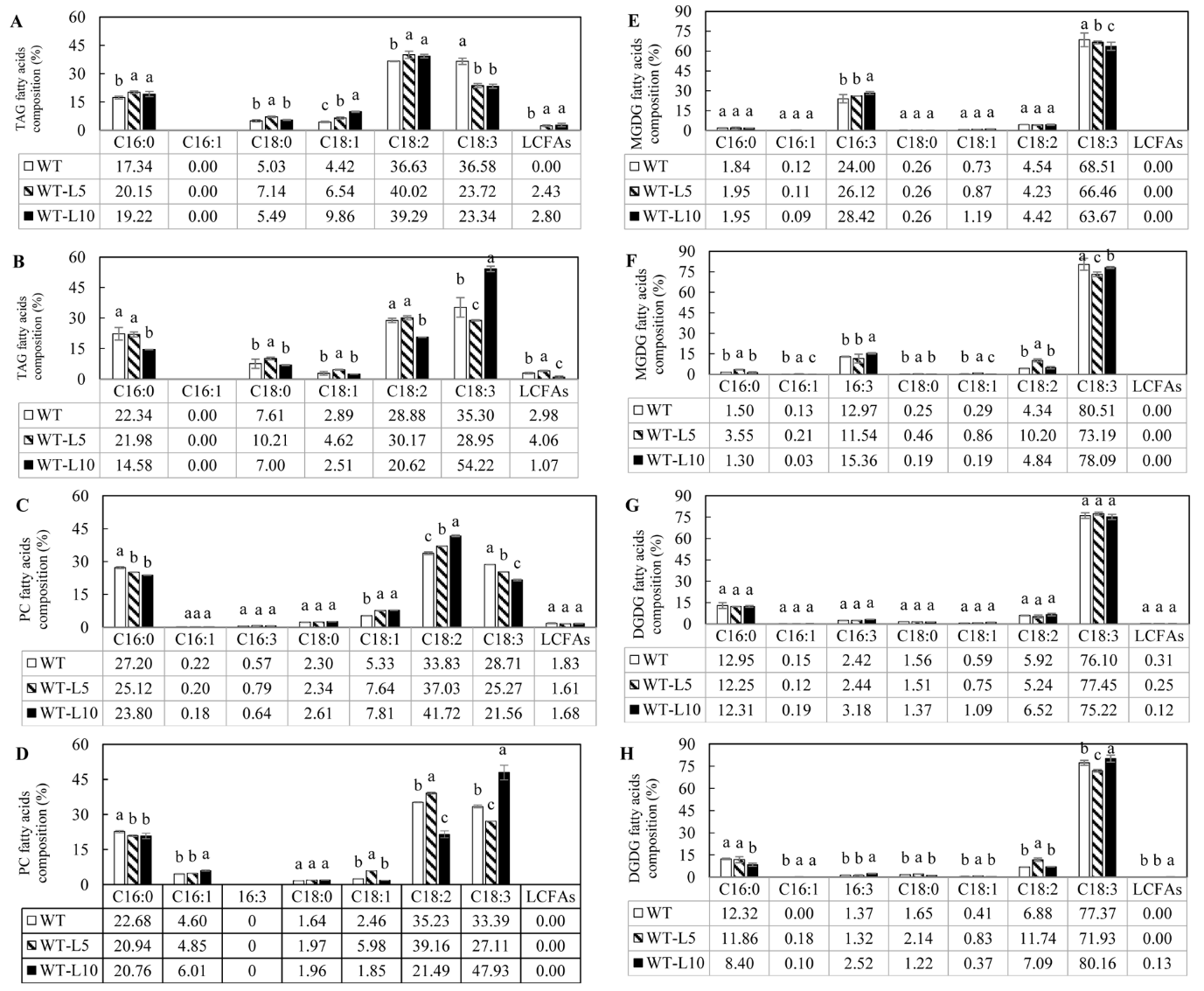


**Supplementary Figure 3.** Fatty acid composition in the lipids of potato leaves of WT (open bars) and the two selected WT-derived lines, WT-L5 (hatched bars) and WT-L10 (black bars) at two developmental stages. (A) TAG at the flowering stage; (B) TAG at the mature stage; (C) PC at the flowering stage; (D) PC at the mature stage; (E) MGDG at the flowering stage; (F) MGDG at the mature stage; (G) DGDG at the flowering stage; (H) DGDG at the mature stage. The data represent the mean values ± SD of three biological replicates. Letters (a, b, c) above the bars are based on LSD, bars marked with different letters are statistically significantly different at P<0.05.


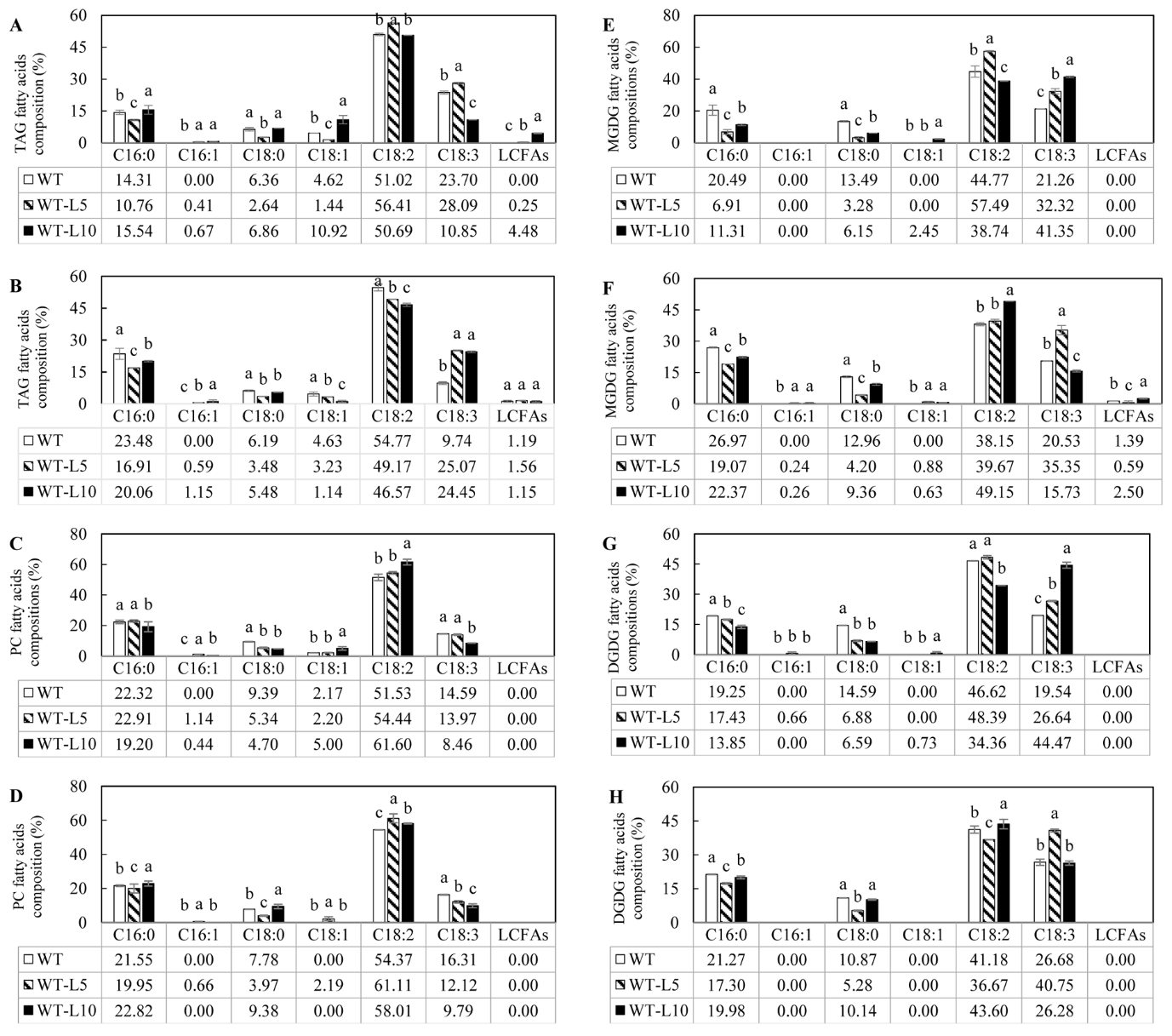


**Supplementary Figure 4.** Fatty acid composition in the lipids of potato tubers of WT (open bars) and the two selected WT-derived lines, WT-L5 (hatched bars) and WT-L10 (black bars) at two developmental stages. (A) TAG at the flowering stage; (B) TAG at the mature stage; (C) PC at the flowering stage; (D) PC at the mature stage; (E) MGDG at the flowering stage; (F) MGDG at the mature stage; (G) DGDG at the flowering stage; (H) DGDG at the mature stage. The data represent the mean values ± SD of three biological replicates. Letters (a, b, c) above the bars are based on LSD, bars marked with different letters are statistically significantly different at P<0.05.


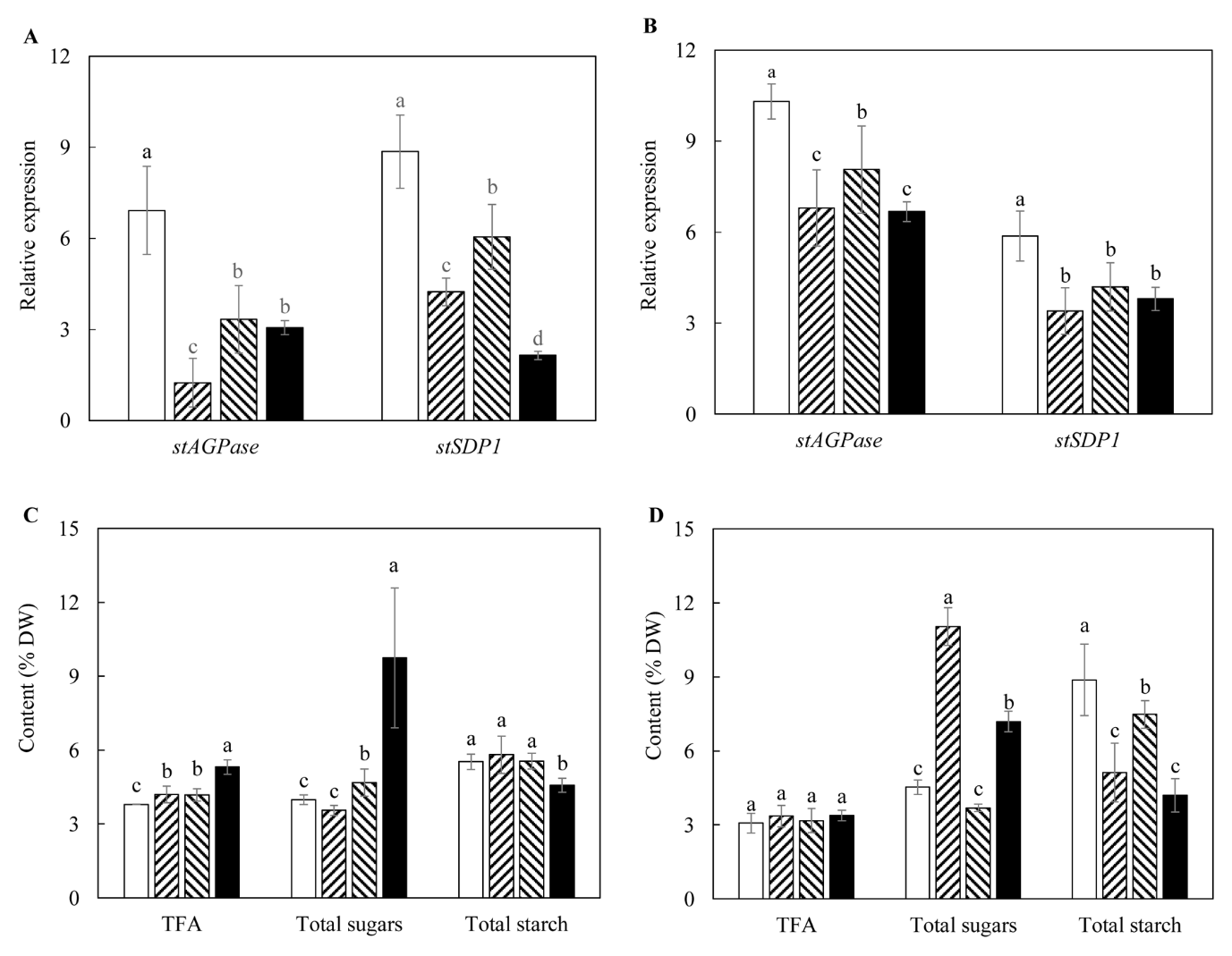


**Supplementary Figure 5.** Gene expression analysis and total carbon allocation in the potato leaves of HO69 (open bars) and three super-transformed lines, 69-L1 (bar with upward trend), 69-L2 (hatched bars) and 69-L3 (black bars) at two developmental stages. (A) Real-time qRT-PCR result at the flowering stage; (B) Real-time qRT-PCR result at the mature stage; (C) Total carbon allocation at the flowering stage; (D) Total carbon allocation at the mature stage. The data represent the mean values ± SD of three biological replicates. Letters (a, b, c) above the bars are based on LSD, bars marked with different letters are statistically significantly different at P<0.05.


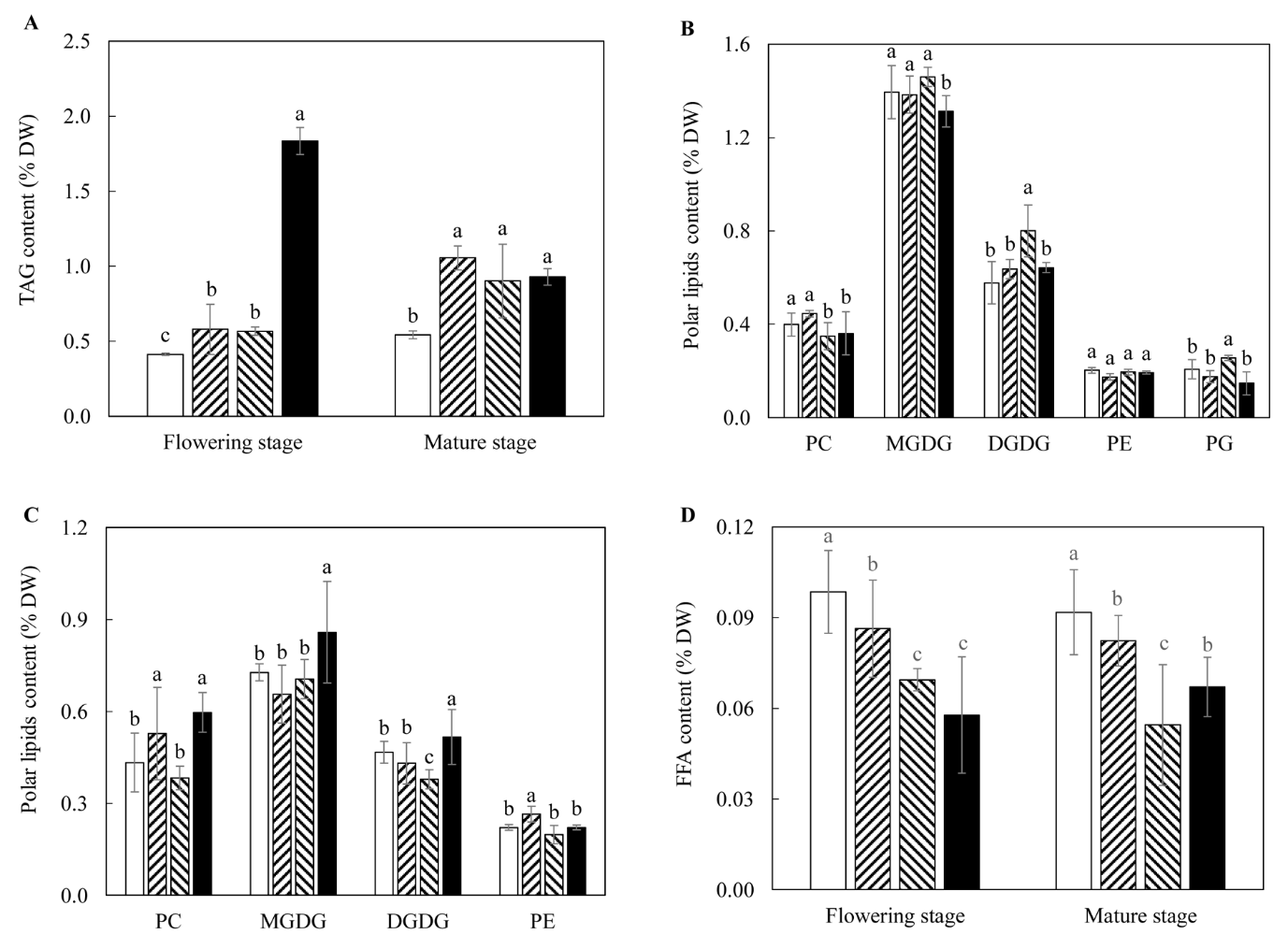


**Supplementary Figure 6.** Contents of TAG, polar lipids and FFA in the potato leaves of HO69 (open bars) and three super-transformed lines, 69-L1 (bar with upward trend), 69-L2 (hatched bars) and 69-L3 (black bars) at two developmental stages. (A) TAG contents; (B) Polar lipids contents at the flowering stage; (C) Polar lipids contents at the mature stage; (D) FFA contents. The data represent the mean values ± SD of three biological replicates. Letters (a, b, c) above the bars are based on LSD, bars marked with different letters are statistically significantly different at P<0.05.


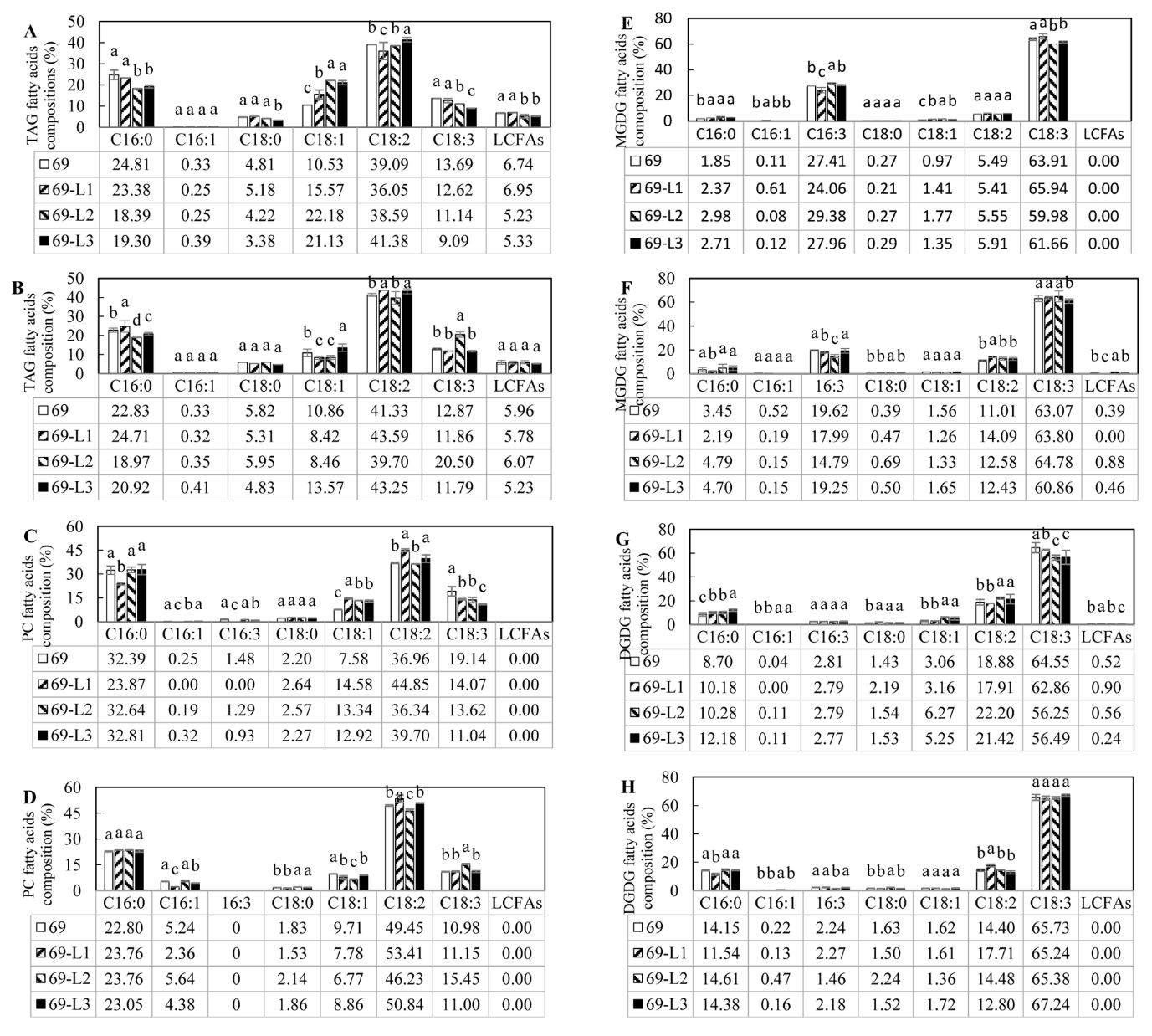


**Supplementary Figure 7.** Fatty acid composition in the lipids of potato leaves of the HO69 (open bars) and three super-transformed lines, 69-L1 (bar with upward trend), 69-L2 (hatched bars) and 69-L3 (black bars) at two developmental stages. (A) TAG at the flowering stage; (B) TAG at the mature stage; (C) PC at the flowering stage; (D) PC at the mature stage; (E) MGDG at the flowering stage; (F) MGDG at the mature stage; (G) DGDG at the flowering stage; (H) DGDG at the mature stage. The data represent the mean values ± SD of three biological replicates. Letters (a, b, c) above the bars are based on LSD, bars marked with different letters are statistically significantly different at P<0.05.


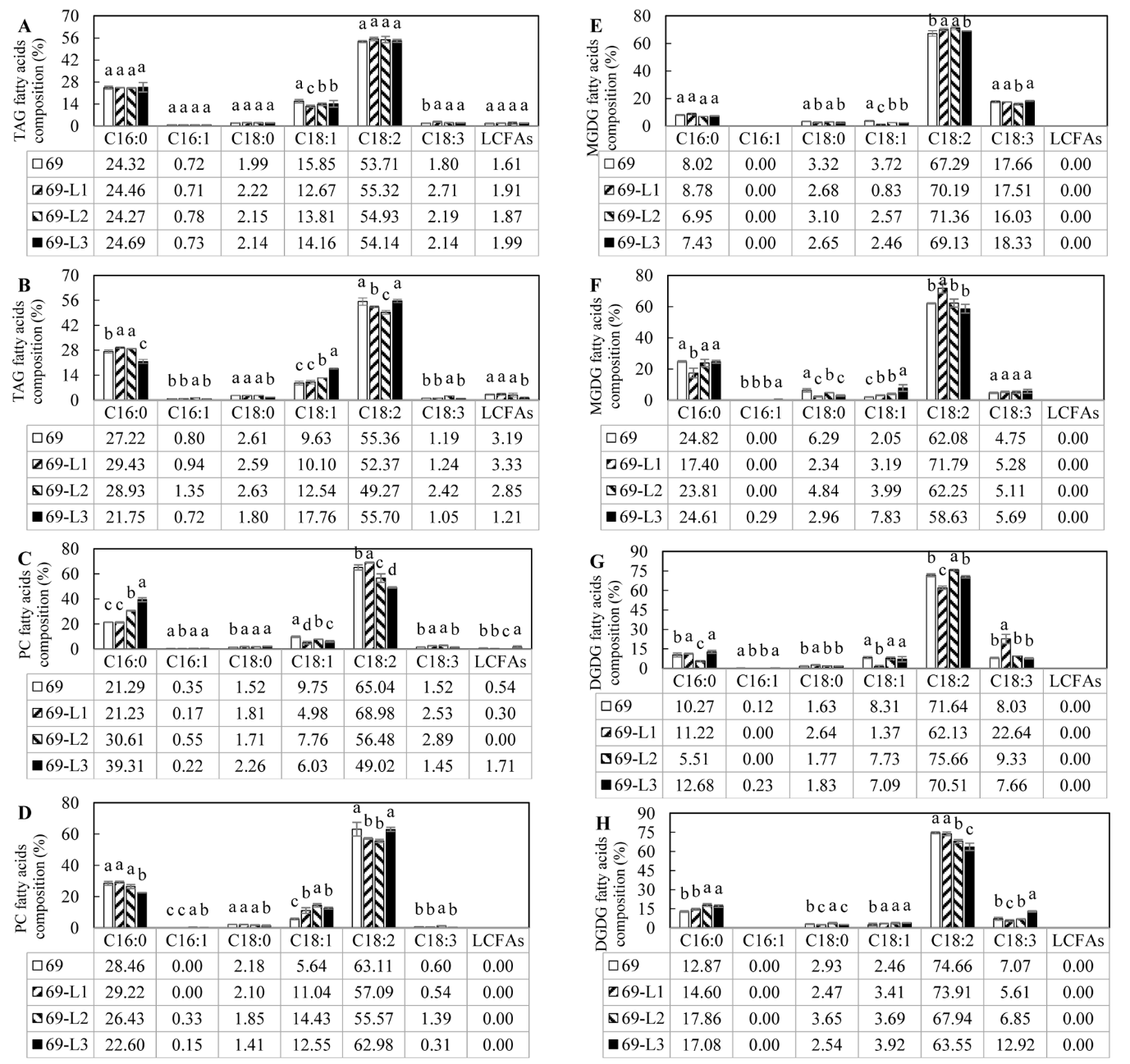


**Supplementary Figure 8.** Fatty acid composition in the lipids of potato tubers of HO69 (open bars) and three super-transformed lines, 69-L1 (bar with upward trend), 69-L2 (hatched bars) and 69-L3 (black bars) at two developmental stages. (A) TAG at the flowering stage; (B) TAG at the mature stage; (C) PC at the flowering stage; (D) PC at the mature stage; (E) MGDG at the flowering stage; (F) MGDG at the mature stage; (G) DGDG at the flowering stage; (H) DGDG at the mature stage. The data represent the mean values ± SD of three biological replicates. Letters (a, b, c) above the bars are based on LSD, bars marked with different letters are statistically significantly different at P<0.05.


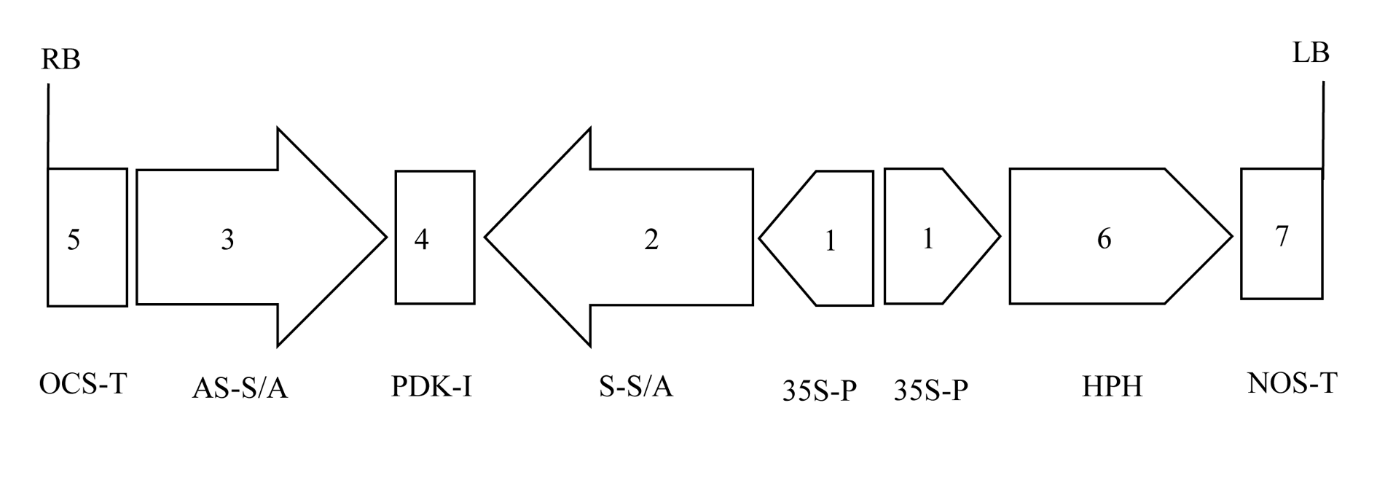
**Supplementary Figure 9.** Configuration of the binary vector pWBVec2-RNAi (*StAGPase*-*StSDP1*) construct. 1, *CaMV-35S* promoter with duplicated enhancer region (35S-P); 2, Sense fragment containing fused regions of *StSDP1*+*StAGPase* (S-S/A); 3, Antisense fragment containing fused regions of *StSDP1*+*StAGPase* (AS-S/A); 4, Pyruvate orthophosphate dikinase intron (PDK-I); 5, Octopine synthase terminator (OCS-T); 6, Hygromycin phosphotransferase (HPH); 7, Nopaline synthase terminator (NOS-T).


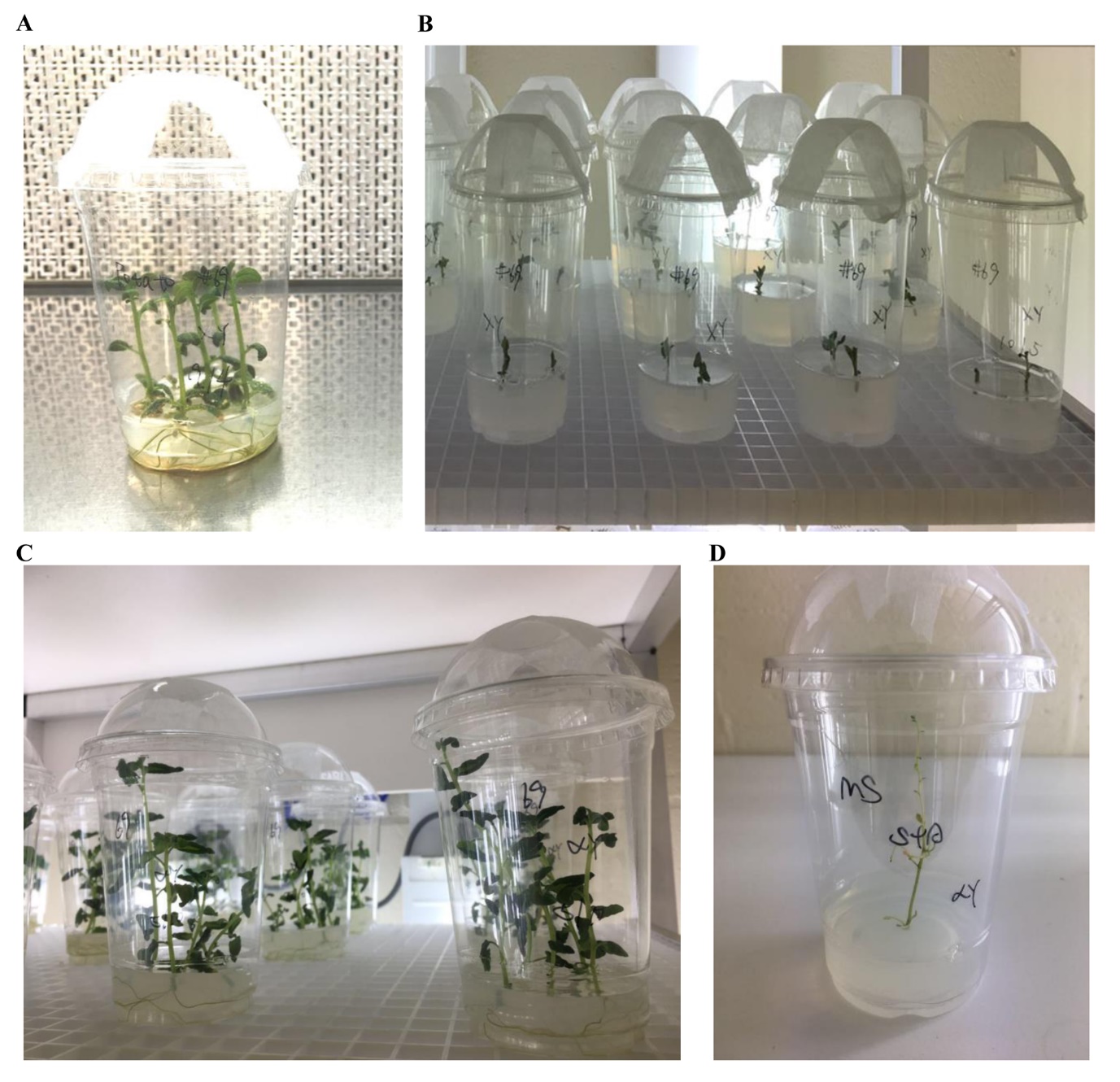


**Supplementary Figure 10.** Establishment of the donor plant system of HO69. (A) Successfully selected aseptic seedlings of HO69; (B-C) Mass propagation of the HO69 aseptic seedlings through the internodes on MS media; (D) Green shoots developed from the calli were transplanted onto the pure MS medium for root induction.

**Supplementary Table 1.** Digital estimation of the protein intensity of the GBPs according to the SDS-PAGE separation

| Samples  Proteins | WT | WT-L5 | WT-L10 |  |
| --- | --- | --- | --- | --- |
|  | Rep1 Rep2 | Rep1 Rep2 | Rep1 Rep2 |  |
| SSII/SBE | 51.35 55.33 | 13.82 55.32 | 29.79 31.25 |  |
| GBSS | 270.02 498.87 | 357.88 582.02 | 500.03 458.30 |  |
| Samples  Proteins | 69 | 69-L1 | 69-L2 | 69-L3 |
|  | Rep1 Rep2 | Rep1 Rep2 | Rep1 Rep2 | Rep1 Rep2 |
| SSII/SBE | 52.89 54.49 | 41.94 45.06 | 50.72 53.42 | 42.83 53.77 |
| GBSS | 165.13 161.01 | 206.04 199.07 | 192.38 191.05 | 139.65 143.66 |

Data represented the intensity values of the major two categories of GBPs, SSII/SBE and GBSS proteins, separated by the SDS-PAGE from the potato tuber starches derived from the WT- and 69-transformations. The layout of data corresponds to their positions marked on the SDS-gel in Fig. 4E & 7G, respectively.

**Supplementary Table 2.** Chain length fractions of WT, line 69 and the three super-transformed lines

|  | 6% ≤ ΣDP ≤ 11% | 12% ≤ ΣDP ≤ 19% | ΣDP > 19% |
| --- | --- | --- | --- |
| WT  Line 69  69-L1  69-L2  69-L3 | 13.28 ± 1.20 ^c^  15.94 ± 1.47 ^a^  14.92 ± 1.07 ^a^  14.55 ± 0.36 ^b^  14.24 ± 0.25 ^b^ | 49.13 ± 1.00 ^a^  50.44 ± 1.22 ^a^  49.77 ± 1.02 ^a^  48.1 ± 0.93 ^b^  50.04 ± 1.06 ^a^ | 37.59 ± 1.14 ^a^  33.62 ± 1.06 ^c^  35.31 ± 1.09 ^b^  37.35 ± 1.08 ^a^  35.72 ± 1.11 ^b^ |

Data represented the mean value of sample ± SD, the same letters followed show no statistically significant difference while those with different letters are statistically significantly different at P < 0.05 between 69 and the three super-transformed lines.
